# A new member of the flavodoxin superfamily from *Fusobacterium nucleatum* that functions in heme trafficking and reduction of anaerobilin

**DOI:** 10.1016/j.jbc.2023.104902

**Published:** 2023-06-10

**Authors:** Alexandra K. McGregor, Anson C.K. Chan, Megan D. Schroeder, Long T.M. Do, Gurpreet Saini, Michael E.P. Murphy, Kirsten R. Wolthers

**Affiliations:** 1 Department of Chemistry, University of British Columbia, Kelowna, Canada; 2 Department of Microbiology and Immunology, Life Sciences Institute, University of British Columbia, Vancouver, Canada

**Keywords:** heme, flavodoxin, flavin, radical SAM, *Fusobacterium nucleatum*

## Abstract

*Fusobacterium nucleatum* is an opportunistic oral pathogen that is associated with various cancers. To fulfill its essential need for iron, this anaerobe will express heme uptake machinery encoded at a single genetic locus. The heme uptake operon includes HmuW, a class C radical SAM-dependent methyltransferase that degrades heme anaerobically to release Fe^2+^ and a linear tetrapyrrole called anaerobilin. The last gene in the operon, *hmuF* encodes a member of the flavodoxin superfamily of proteins. We discovered that HmuF and a paralog, FldH, bind tightly to both FMN and heme. The structure of Fe^3+^-heme–bound FldH (1.6 Å resolution) reveals a helical cap domain appended to the ⍺/β core of the flavodoxin fold. The cap creates a hydrophobic binding cleft that positions the heme planar to the *si*-face of the FMN isoalloxazine ring. The ferric heme iron is hexacoordinated to His134 and a solvent molecule. In contrast to flavodoxins, FldH and HmuF do not stabilize the FMN semiquinone but instead cycle between the FMN oxidized and hydroquinone states. We show that heme-loaded HmuF and heme-loaded FldH traffic heme to HmuW for degradation of the protoporphyrin ring. Both FldH and HmuF then catalyze multiple reductions of anaerobilin through hydride transfer from the FMN hydroquinone. The latter activity eliminates the aromaticity of anaerobilin and the electrophilic methylene group that was installed through HmuW turnover. Hence, HmuF provides a protected path for anaerobic heme catabolism, offering *F. nucleatum* a competitive advantage in the colonization of anoxic sites of the human body.

The Gram-negative anaerobe, *Fusobacterium nucleatum*, is a prominent member of the oral microbiome. Although *F. nucleatum* is considered an oral commensal, metabolic and physical attributes of the bacterium implicate it in periodontal disease. For example, in the subgingival layer, the bacterium acts as a physical “bridge” between early colonizers (*e.g.*, aerotolerant streptococci) and late colonizers that includes anaerobic periodontal pathogens (1). Coaggregation of other anaerobes with *F. nucleatum* also protects them from oxidative stress (2). A more recent study showed that polyamine formation by *F. nucleatum*—supported by metabolite cross-feeding from early colonizers—enhances the biofilm life cycle of the keystone pathogen, *Porphyromonas gingivalis* (3). Interest in the pathogenic nature of *F. nucleatum* beyond that of oral infections has escalated in the past decade due to the bacterium’s prevalence in tumors of the colon, breast, esophagus, and pancreas (4, 5, 6, 7, 8). Studies have shown that *F. nucleatum* can disseminate hematologically and invade a broad range of host cell types, inducing an inflammatory response (9, 10). The pervasiveness of the bacterium at cancerous sites is associated with tumor progression (11, 12, 13), chemotherapeutic resistance (14), induced metastasis (8), and poorer patient prognosis (14, 15, 16, 17).

The emerging pathogenic nature of *F. nucleatum* merits further investigation into mechanisms by which the organism is able to acquire essential nutrients. One of these essential nutrients is iron, which has shown to be a limiting factor in the ability of pathogens to infect and colonize the host (18). Considering that the vast majority (>95%) of iron in vertebrates is sequestered in heme-containing proteins, it is unsurprising that many bacteria evolved sophisticated mechanisms for acquiring and degrading heme to satisfy their iron requirement. In Gram-negative bacteria, the import of heme involves a high-affinity TonB-dependent receptor in the outer membrane (HmuR), an ABC-type transport system in the inner membrane (HmuUV), and a periplasmic shuttle protein (HmuT) (19, 20, 21). *F. nucleatum* subsp. *nucleatum* ATCC 25586 encodes heme transport machinery at a single locus (*hmuTRUV*; FN0767-FN0770; Fig. 1), which was upregulated following a decrease in cellular iron levels caused by exposure of the bacterial culture to a zinc ionophore (22). Transcriptome analysis further showed upregulation of *hmuR* in *F. nucleatum* isolated from the periodontal cavity in comparison to laboratory culture (23). These results signify that the heme uptake system is important in maintaining iron homeostasis under iron duress, which may occur in a polymicrobial population.

**Figure 1:**
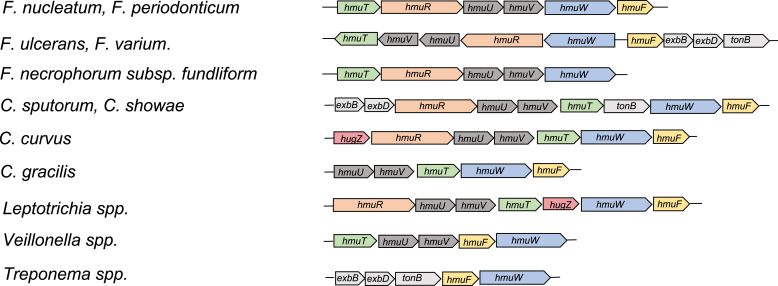
**Heme uptake/utilization operons of various Gram-negative bacteria.** Heme uptake gene clusters showing the common colocalization of genes encoding HmuF and HmuW (anaerobilin synthase) in *Fusobacterium nucleatum* and other Gram-negative bacteria including other *Fusobacteria* (*Fusobacteria peridonticum*, *Fusobacteria ulcerans, Fusobacteria varium*), several oral associated species of *Campylobacter (Campylobacter sputorum, Campylobacter showae, Campylobacter curvus,* and *Campylobacter gracilis*), *Leptotrichia* (*Leptotrichia trevisanii*, *Leptotrichia hofstadii*, and *Leptotrichia hongkongensis*), *Veillonella* (*Veillonella dispar* and *Veillonella atypica*), and *Treponema* (*Treponema denticola* and *Treponema pedis*). Other gene products in the clusters include HmuT (periplasmic heme-binding protein), HmuR (TonB-dependent outer membrane heme receptor), HmuU (inner membrane permease), HmuV (cytoplasmic ATPase), exbB and exbD (subunits of the TonB-dependent energy transduction system), TonB (the energy transducer), and HugZ (heme oxygenase).

Two contiguous genes at this locus are FN0771 and FN0772, which we have termed *hmuW* and *hmuF*, respectively. The latter gene is annotated as a flavodoxin, while the former gene encodes a protein homologous to ChuW/HutW, a class C radical SAM-methyltransferase (RSMT) involved in the anaerobic degradation of heme. First discovered in the enteric pathogens *Escherichia coli* O157:H7 and *Vibrio cholerae*, ChuW/HutW, also known as anaerobilin synthase, catalyzes the methylation of a sp^2^ carbon of the tetrapyrrole ring. This reaction causes decyclization of the macrocycle and release of Fe^2+^ (24). As with all radical SAM enzymes, the catalytic cycle initiates with donation of an electron from the embedded [Fe_4_S_4_]^1+^ redox cluster to a bound SAM molecule. This electron transfer step leads to reductive cleavage of the SAM C-S bond and formation of a 5′-deoxyadenosyl radical (Ado·). In class C RSMT, the highly reactive Ado· abstracts a hydrogen atom from the methyl group of a second SAM molecule, generating a methylene radical which adds to a *meso* carbon of the porphyrin ring. In the proposed reaction mechanism (Fig. 2), protonation of a neighboring pyrrole leads to β-scission of the ring and elimination of SAH (25). Further hydride transfer or proton-coupled electron transfer quenches the radical on the porphyrin. The resulting linearized tetrapyrrole, termed anaerobilin, is potentially toxic due to its aromaticity and the presence of a newly installed methylene group, which serves as a good Michael acceptor to free thiols.

**Figure 2:**
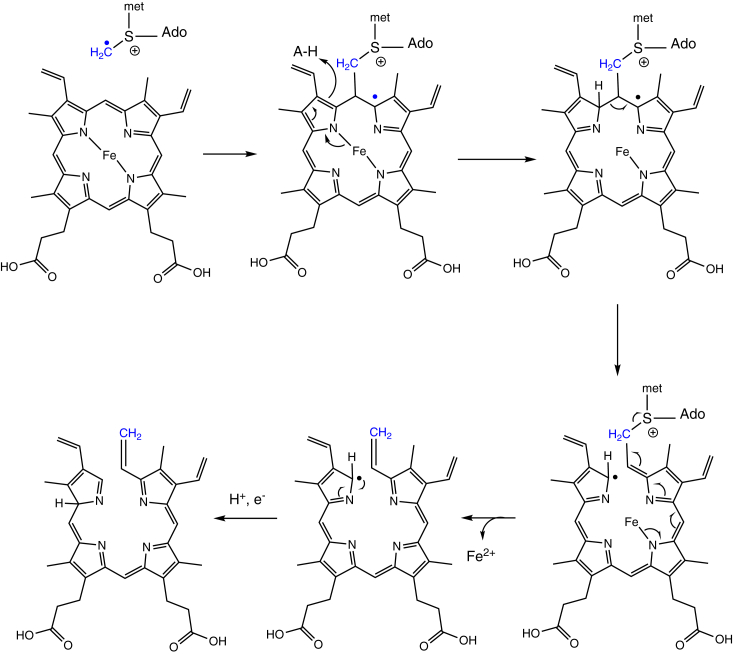
**Proposed reaction mechanism for HmuW**.

In the radical SAM superfamily of enzymes, flavodoxin reduces the [4Fe-4S]^+2^ to [4Fe-4S]^+1^ to facilitate another round of catalysis. We were intrigued by the presence of *hmuF* in the heme acquisition gene cluster and wondered if this putative flavodoxin elicited a high level of specificity for HmuW compared to other flavodoxins encoded in the *F. nucleatum* genome. To our surprise, purification of recombinant HmuF and a paralog FldH (FN1822) bind tightly to Fe^3+^-hemin in addition to the FMN cofactor. Herein, we show that HmuF does not function in single electron transfer as a typical flavodoxin. Instead, HmuF binds heme and traffics it to HmuW for radical-mediated decyclization, and then it functions as an anaerobilin reductase. In this dual role, HmuF mitigates against the toxicity of labile heme and anaerobilin.

## Results

### Genetic analysis

The *hmuF* gene in *F. nucleatum* subsp. *nucleatum* ATCC 25586 is located at the end of the *hmu* gene cluster (*hmuTRUVWF*), whereas *fldH* is monocistronic. To gain insight into the putative functional role of HmuF in the heme uptake/utilization pathway, close homologs (>40% sequence identity) of HmuW were searched for in the NCBI database. Analysis of the genomes of close homologs revealed that the *hmu* cluster is conserved in several *Fusobacterium* species that inhabit the oral cavity, including the three other subspecies of *F. nucleatum* (*polymorphum*, *animalis* and *vicentii*) and the closely related species *Fusobacterium periodonticum* (Fig. 1). Two other species of *Fusobacteria* that form a distinct lineage (26), *Fusobacterium ulcerans* and *Fusobacterium varium*, also contain a similar operon, but *hmuF* is transcribed in the opposite direction from *hmuW*. *Fusobacterium necrophorum* subsp. *funduliform*; the causative agent of Lemierre’s syndrome contains the heme acquisition operon with *hmuW*, but lacks *hmuF* (27). Similar heme uptake/utilization operons were identified in oral-associated species of *Campylobacter*, *Leptotrichia* (*Leptotrichia trevisanii*, *Leptotrichia hofstadii*, and *Leptotrichia hongkongensis*), *Veillonella* (*Vibrio dispar* and *Vibrio atypica*), and *Treponema* (*Treponema denticola* and *Treponema pedis*). Given their close association in the oral biofilm, it is conceivable that these organisms acquired the gene cluster through horizontal gene transfer. Although the gene clusters show the absence or presence of different genes in the iron uptake/utilization pathway, *hmuW* and *hmuF* are often contiguous. Given the frequent colocalization of *hmuW* and *hmuF*, it is reasonable to conjecture that the two proteins act together in the anaerobic metabolism of heme.

### Purification and UV-visible absorbance properties of FldH and HmuF

**Figure 3:**
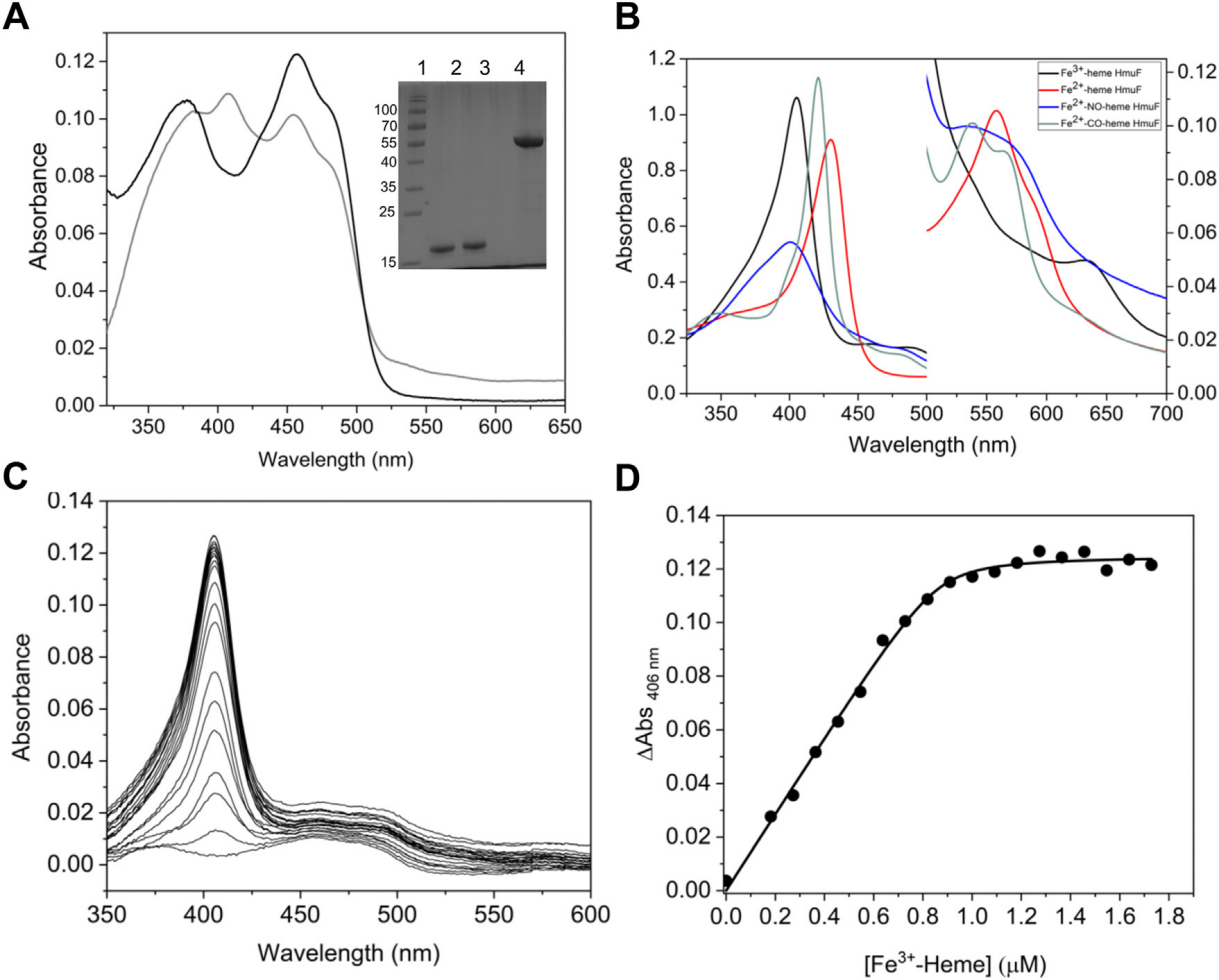
**HmuF spectral properties and heme-binding assays.***A*, UV-visible spectra of HmuF following Ni-NTA (*gray line*) and ion exchange chromatography (*black line*). Inset: Homogeneity of protein preparations determined by 12% SDS-PAGE. MW markers (Lane 1: *top to bottom*, 180 kDa, 130 kDa, 100 kDa, 70 kDa, 55 kDa, 40 kDa, 35 kDa, 25 kDa, 15 kDa), purified FldH (lane 2), HmuF (lane 3), and HmuW (lane 4). *B*, UV–visible absorption spectra of heme-bound HmuF in different ligation and redox states (*black line*, Fe^3+^-heme; *gray line*, Fe^2+^-heme; *blue line*, NO-Fe^2+^ heme; *red line*, CO-Fe^2+^ heme). *C*, heme titration of HmuF. A Fe^3+^-hemin solution was simultaneously titrated into a 1 ml solution of 0.9 μM FldH in 100 mM Hepes, pH 7.0 and a reference cuvette containing buffer only. The absorbance spectra were recorded after each addition of hemin. *D*, the change in absorbance at 406 nm for each titration point was plotted as a function of heme concentration, and the data were fitted to the quadratic binding isotherm (eq 1) which gave a *K*_d_ of 9.1 ± 4.7 nM. NTA, nitrilotriacetic.

The recombinant forms of FldH and HmuF (38.5% sequence identity) were expressed in *E. coli* BL21(DE3)pLysS. Upon elution from the Ni-nitrilotriacetic (NTA) column, both proteins were amber, not the typical bright yellow associated with purified flavodoxin. The UV-visible absorption spectrum of HmuF elicited peaks at 377 and 456 nm with a shoulder at 480 nm, typical of a protein-bound FMN cofactor (Fig. 3*A*). The additional peak at 406 nm in the spectrum suggested substoichiometric amounts of heme present in the protein sample. FldH also displayed a ƛ_max_ at 406 nm following elution from the Ni-NTA column, but the FMN absorbance peaks (384 and 459 nm) were more red-shifted in comparison to HmuF, and a prominent shoulder appeared at 484 nm (Fig. S1). Application of HmuF to a Q-Sepharose column removed detectable traces of the heme: the eluted protein was yellow and the UV-visible absorbance spectrum only shows FMN absorbance peaks at 377 and 456 nm (Fig. 3*A*). In contrast, heme remained bound to FldH following ion exchange chromatography, suggesting that the heme cofactor is more tightly coordinated to this homolog. A 12% SDS-polyacrylamide gel of recombinant HmuF and FldH showed that the samples were homogenous and had apparent molecular masses of 20 kDa, similar to the calculated molecular masses of 19.3 kDa (Fig. 3*A*, inset). The calculated extinction coefficient of the FMN cofactor bound to HmuF is 11,515 M^−1^ cm^−1^.

### Electronic absorption spectra of heme-bound HmuF and FldH

To determine the heme coordination environment of HmuF and FldH, 1.2 M equivalents of hemin were added to purified HmuF or FldH and unbound hemin was removed by applying both proteins to a PD10 desalting column. The resulting Fe^3+^-heme–bound forms of HmuF and FldH elicited Soret peaks at 406 nm (Figs. 3*B* and S2). A peak at 628 nm was also observed for heme-bound HmuF, while a broad band at 574 nm with a shoulder at 630 nm appeared in the spectrum of heme-bound FldH. The electronic absorption spectra of both proteins indicate that the ferric heme iron state is 6-coordinate high spin (28). Reduction of the heme with sodium dithionite shifts the Soret maximum to 429 nm and 424 nm for HmuF and FldH, respectively, and results in a sharp ⍺/β band with a ƛ_max_ at 558 nm for both proteins. These spectral features are indicative of a 5-coordinate low spin iron. The addition of CO converts both HmuF and FldH to a 6-coordinate low spin iron species evidenced by a blue shift in the Soret band with a ƛ_max_ at 421 nm and a splitting of the ⍺ and β bands. Intriguingly, the addition of NO to the ferrous heme results in a 5-coordinate ferrous–nitrosyl–iron complex evidenced by the large blue shift in the Soret band with a ƛ_max_ at 401 nm. A six-coordinate complex is expected to have a Soret band with the maximum centered at 421 nm (29). There is also splitting of the ⍺ and β bands, which is more muted than that of CO-heme HmuF–FldH complexes. Thus, NO binding ruptures the bond between the Fe^2+^ and the axial ligand, as also observed in the NO-sensor soluble guanylate cyclase, its bacterial homolog, the heme-nitric oxide/oxygen binding proteins, cytochromes c', and the O_2_ sensor protein Fix-L (30, 31). A summary of the ƛ_max_ values for the different heme ligation states and the extinction coefficients for the Soret bands is presented in Table S1.

### Heme binding

Difference spectroscopy was used to quantitate the binding affinity of hemin to HmuF. We could not separate heme from FldH without also causing the loss of FMN and protein precipitation. Consequently, heme-binding studies were only performed for HmuF devoid of heme following ion-exchange chromatography. When 0.9 μM HmuF was titrated with Fe^3+^-hemin, the difference spectra exhibited absorbance changes in the Soret region at 406 nm (Fig. 3*C*). A plot of the absorbance change at 406 nm *versus* the concentration of added Fe^3+^-hemin showed absorbance saturation at ∼1 μM of added hemin, suggesting that HmuF binds one equivalent of heme per polypeptide (Fig. 3*D*). A fit of the data to a quadratic-binding isotherm generated a *K*_d_ value of 9.1 ± 4.7 nM. This *K*_d_ value is viewed as an upper limit for the dissociation constant of heme due to the fact that near micromolar concentrations of Fe^3+^-hemin and HmuF were added to the titrations. Under these conditions, there is no free hemin in the titration as most of it is bound to the protein (32).

### Redox properties of the FMN and heme cofactors

**Figure 4:**
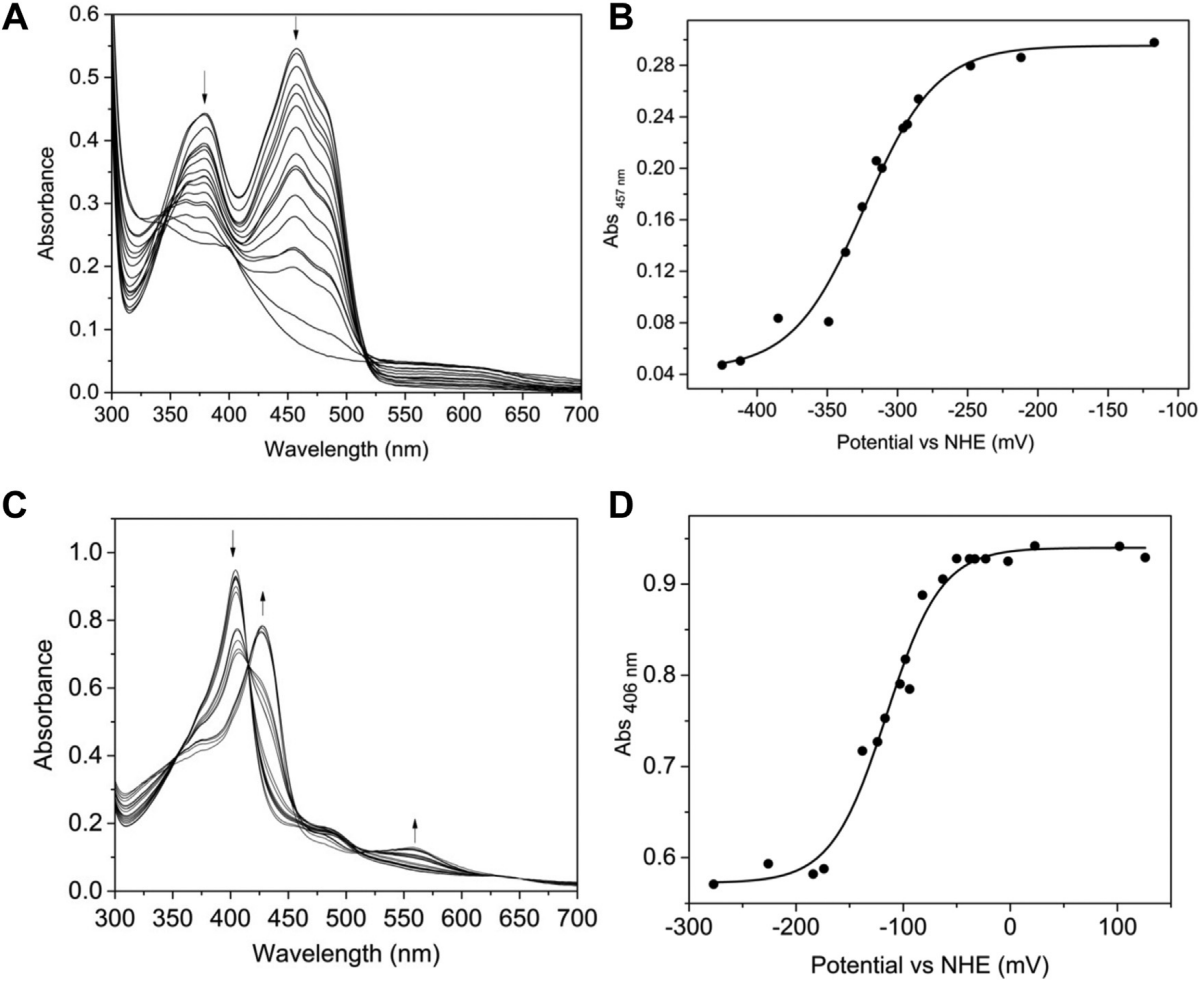
**Chemical redox titrations of HmuF.** Absorption spectra for the redox titration of the HmuF. Electronic absorption spectra of (*A*) HmuF and (*C*) heme-bound HmuF acquired after each addition of dithionite. *Arrows* indicate direction of absorbance change. Plots of absorbance *versus* potential *versus* the normal hydrogen electrode (NHE) for (*B*) HmuF, (*D*) heme-bound HmuF. Data in panels (*B* and *D*) were fitted to eq 3.

The reduction potentials of protein-bound FMN and heme cofactors were determined by measuring the absorbance spectra of the protein as it was chemically reduced with sodium dithionite. HmuF (free of heme) was introduced into the glove box and exchanged into anaerobic buffer as described in Experimental procedures. Successive reduction of the oxidized FMN cofactor with sodium dithionite resulted in gradual bleaching of the flavin absorbance maxima at 384 and 459 nm (Fig. 4*A*). In contrast to flavodoxins, reduction of the protein did not lead to a transient absorbance band at ∼590 nm that would otherwise indicate formation of the blue neutral semiquinone. A red anionic semiquinone (ƛ_max_ at 390 nm) signal was also absent in the dithionite titration. These data indicate that HmuF participates in hydride transfer reactions as opposed to single electron transfers. For HmuF, the absorbance change at 454 nm was plotted against the observed potential and a fit of the data to equation 1 yielded a reduction potential of −323 ± 4 mV *versus* the normal hydrogen electrode (Fig. 4*B*). Chemical reduction of the heme-bound forms of HmuF and FldH resulted in conversion of ferric to ferrous heme noted by the shift in the ƛ_max_ of the heme cofactor (Figs. 4*C* and S3). Figure 4*D* shows the absorbance at 406 nm *versus* the measured potential for heme; a fit of equation 3 to the data yielded reduction potential of −115 ± 4 mV for the Fe^3+^/Fe^2+^-heme couple. A similar reduction potential was obtained for FldH (Fig. S3).

### Enzymatic reduction of HmuF and heme-loaded HmuF

**Figure 5:**
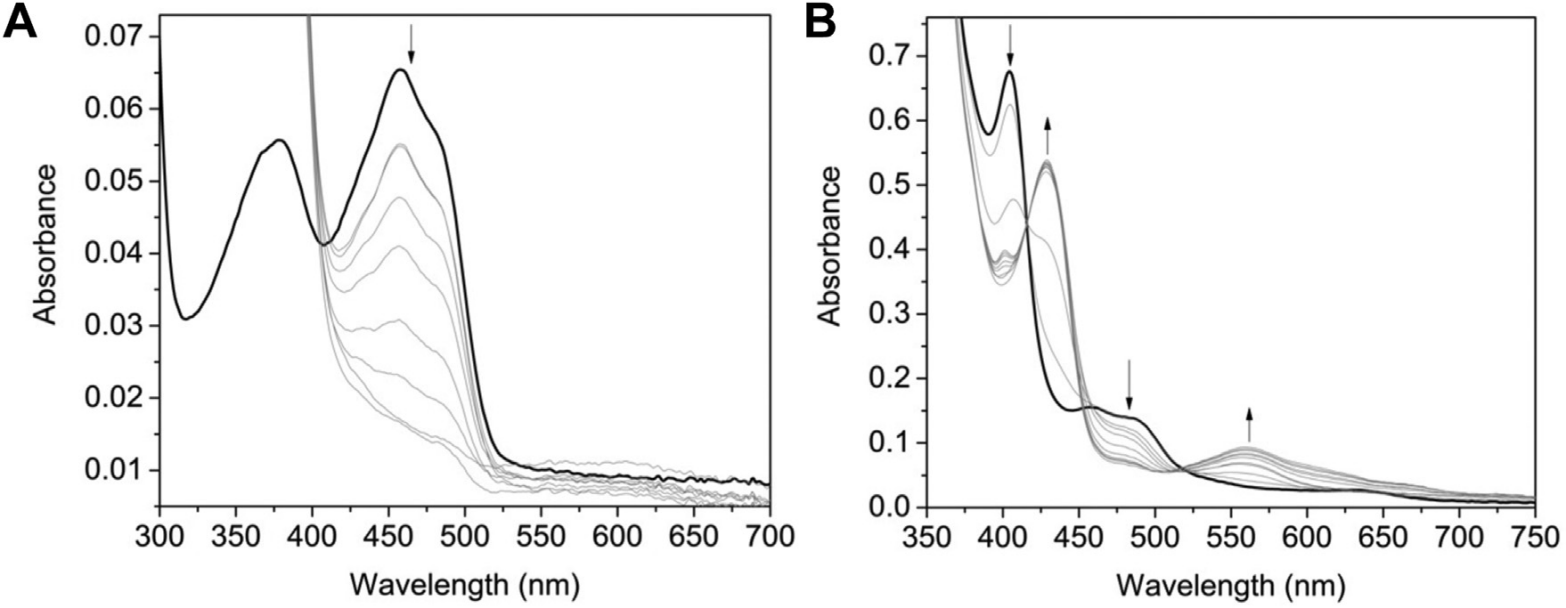
**NADPH-dependent reduction of HmuF and heme-loaded HmuF by aaCPR**_**FAD**_**.** UV-visible absorbance changes following addition of 0.5 μM aaCPR, 250 μM NADPH to (*A*) 10 μM HmuF and (*B*) 10 μM of Fe^3+^-heme-HmuF. Reactions were initiated with the addition of NADPH. The *black line* in both panels is 10 μM HmuF and 10 μM of Fe^3+^ heme-HmuF prior to the addition of NADPH, and *gray lines* are after addition of the reduced pyridine nucleotide. In panels (*A* and *B*), spectra were acquired ∼10 s following first addition of NADPH and then every 10 min (*A*) or 2 min (*B*) until no further spectral changes were observed.

We next determined if HmuF could be reduced to the hydroquinone state through electron transfer from an NAPDH-dependent flavodoxin reductase (FprA). *F. nucleatum* does not have an FprA homolog; therefore, we used a truncated form of a cytochrome P450 reductase from *Artemisia annua* (aaCPR_FAD_) that contains the NADPH and FAD-binding modules but is missing the C-terminal FMN-binding flavodoxin domain. As shown in Figure 5*A*, aaCPR_FAD_ catalyzes the NADPH-dependent reduction of HmuF under anaerobic conditions. Similar to the chemical reduction with dithionite, the FMN semiquinone did not form during the transition from oxidized to hydroquinone states. The addition of aaCPR_FAD_ and NAPDH to the Fe^3+^ heme–HmuF complex caused reduction of the ferric heme to the ferrous form as evidenced by a shift in the Soret maximum from 406 to 429 nm and the appearance of a β-band at 570 nm. There is also a decrease in absorbance between 460 to 520 nm, which likely signifies the reduction of the HmuF FMN cofactor to the hydroquinone state. Complete reduction of the FMN cofactor was slower than the heme, likely due to its more electronegative reduction potential. NADPH alone did not lead to reduction of either the FMN or heme. aaCPR_FAD_ could also catalyze the NADPH-dependent reduction of the FMN and iron in heme-loaded FldH (Fig. S4).

### HmuF traffics heme to HmuW and catalyzes reduction of anaerobilin

**Figure 6:**
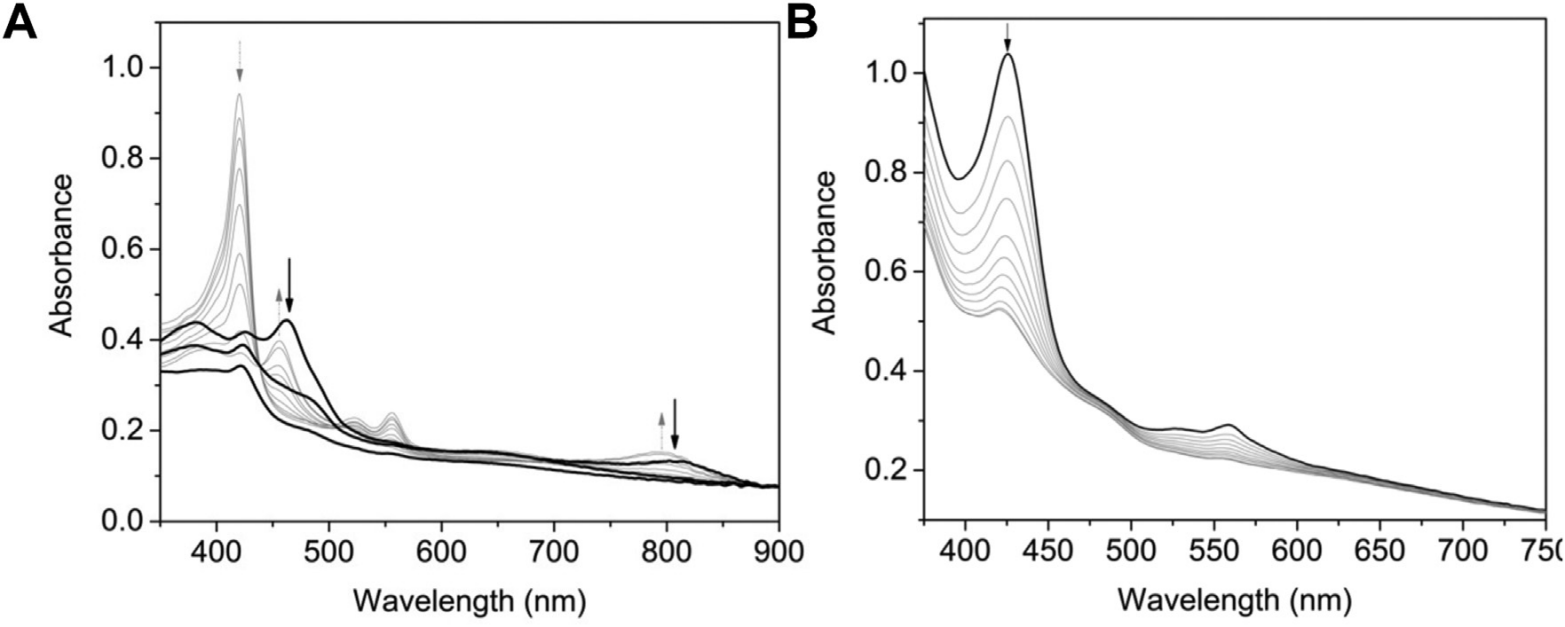
**Heme trafficking and anaerobilin reduction by HmuF.***A*, UV-visible spectroscopic assays were performed by adding HmuW (2 μM) to a solution containing 10 μM hemin, 2 μM aaCPR_FAD_, 2 μM FnFld, 200 μM NADPH. The reaction was initiated with the addition of 250 μM SAM. The spectra were acquired every 2 min for 45 min (*gray lines*). The *arrows* indicate the direction of the absorbance change. Once anaerobilin was formed, denoted by no further absorbance changes at 795 nm; HmuF (5 μM) was added to the reaction. The *black lines* denote absorbance changes in anaerobilin at various time points. *B*, absorbance spectrum of 10 μM Fe^2+^-heme–bound HmuF in a solution containing 2 μM aaCPR_FAD_, 2 μM FnFld, 200 μM NADPH (*black line*). Absorbance changes in the heme Soret band following subsequent addition of HmuW (2 μM) and 250 μM SAM (*gray lines*). Spectra were acquired every 5 min for 45 min.

To determine if HmuF is involved in heme degradation in concert with HmuW, we first cloned, expressed, and purified HmuW as described in the Experimental procedures. SDS-PAGE analysis revealed a homogeneous protein sample (Fig. 3*A*, inset), and reconstitution with iron and sulfur generated 3.52 ± 0.26 and 3.76 ± 0.18 atom equivalents, respectively per polypeptide. Prior to performing experiments with HmuF, we assayed for anaerobilin formation by HmuW through UV-visible spectrophotometry, as described by LaMattina *et al.* (24). A previously characterized flavodoxin produced by *F*. *nucleatum*, FnFld, was used as the one electron donator to the [Fe_4_S_4_]^2+^ cluster of HmuW (33). UV-visible spectroscopic assays confirmed that aaCPR_FAD_ could efficiently reduce FnFld to the hydroquinone state *via* a semiquinone intermediate (Fig. S5). In a reaction containing NAPDH, aaCPR_FAD_, FnFld, HmuW, hemin, SAM, and 5% v/v dimethyl sulfoxide, we observed a decrease in the heme Soret band at 420 nm and an increase in absorbance at 445 and 795 nm, indicating anaerobilin formation (Fig. 6*A*). As reported for ChuW, the HmuW RSMT reaction is slow, possibly due to insolubility of the anaerobilin product. HmuW could also catalyze ring opening of deuteroheme, a more soluble form of the cofactor (Fig. S6).

Following conversion of heme to anaerobilin or deuteroheme to deuteroanaerobilin (judged by no further absorbance changes at 445 and 795 nm), HmuF was added to the reaction mixture. As presented in Figure 6*A* and Fig. S6, HmuF addition triggered an immediate (<30 s) red-shift in the anaerobilin and deuteroanaerobilin peaks to a ƛ_max_ of 450 and 805 nm, suggesting that HmuF binds to the linearized tetrapyrroles. Spectra acquired 5 min and 10 min later show a decrease in the anaerobilin and deuteroanaerobilin peaks, indicating a loss in conjugation in the ring system possibly through reduction of the linearized tetrapyrroles. A repeat of the experiment with FldH instead of HmuF resulted in similar changes in the anaerobilin spectra (Fig. S7).

Anaerobic UV-visible spectroscopy was also used to determine if HmuW could catalyze anaerobic degradation of the heme cofactor when coordinated to HmuF. In this experiment, HmuF (10 μM) was mixed with an equimolar concentration of hemin to form the Fe^3+^-heme–bound HmuF. The addition of NAPDH, aaCPR_FAD_, and FnFld to the reaction mixture led to reduction of Fe^3+^-heme to Fe^2+^-heme and reduction of the oxidized FMN to the hydroquinone (FMNH^−^). The resulting absorbance spectrum of Fe^2+^ -heme-HmuF elicited a maximum at 429 nm (Fig. 6*B*). The subsequent addition of HmuW (2 μM) and SAM (250 μM) lead to a decrease in the Soret peak and the β-band at 560 nm. These spectral changes indicate degradation of the heme cofactor likely through HmuW radical–mediated decyclization followed by reduction of the tetrapyrrole ring. A repeat of the experiment with FldH instead of HmuF resulted in similar changes in the Soret band (Fig. S7).

### MS analysis of reaction products

**Figure 7:**
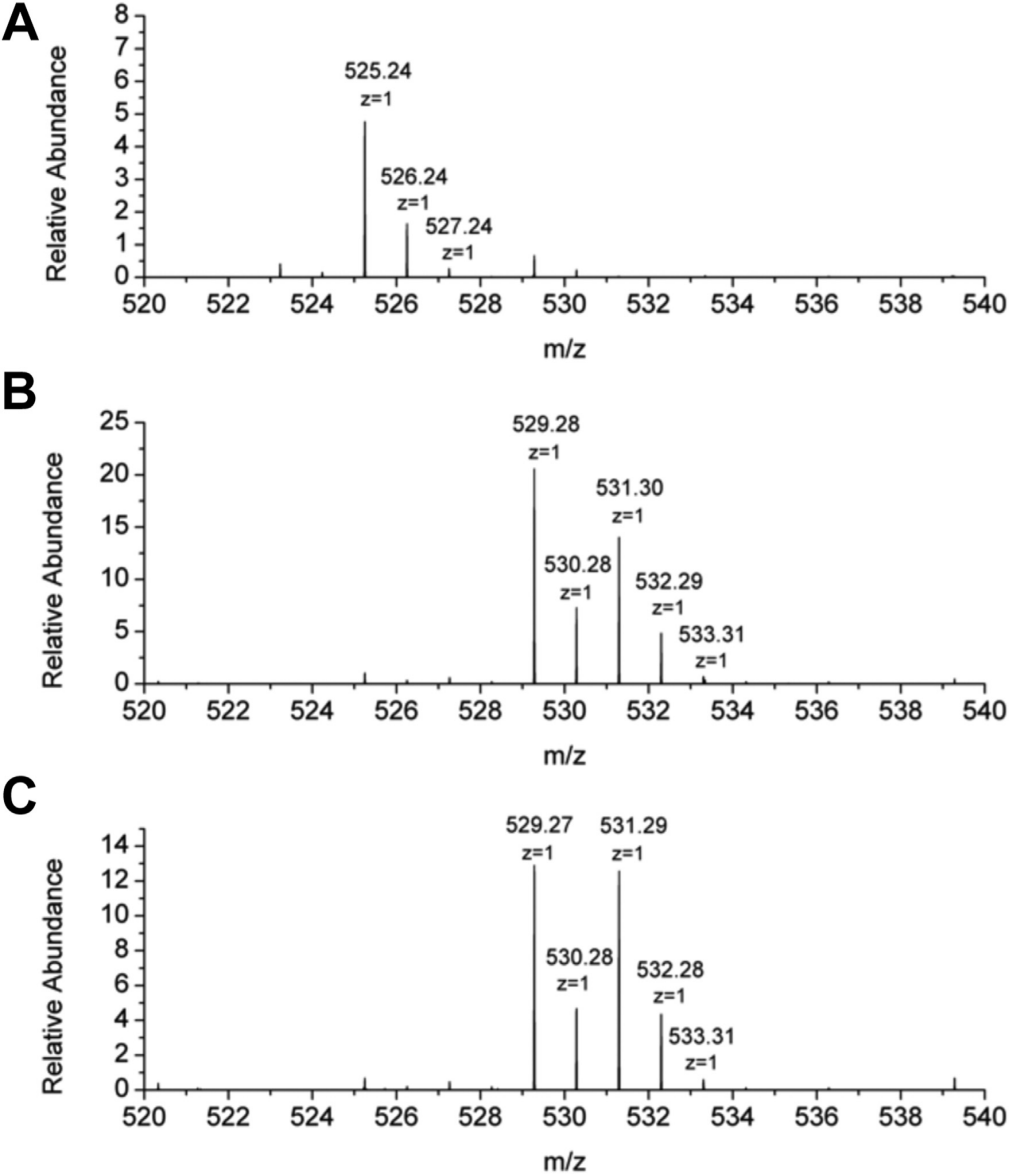
**MS of linearized tetrapyrrole products.** (*A)* deuteroanaerobilin generated in a 5 ml reaction containing 10 μM HmuW, 400 μM SAM SAM, 5 μM aaCPR_FAD_, 5 μM FnFld, 500 μM NADPH, and 20 μM deuteroheme (*B*) 5 ml reaction as for (*A*), except with the addition of 10 μM HmuF following the conversion to deuteroanaerobilin by HmuW, and (*C*) 5 ml reaction as for (*A*), except with the addition of 10 μM FldH following the conversion to deuteroanaerobilin.

Mass spectrometry (MS) was used to determine if HmuF was functioning as an anaerobilin reductase through hydride transfer to the tetrapyrrole ring. When the RSMT reaction was performed in the absence of HmuF or FldH, we observed a dominant peak at m/z = 525.24 [M + H]^+^, which is equal to the mass of deuteroporphyrin with an additional CH_2_ group (Fig. 7). We observed a second peak at m/z = 526.24 [A + 1] that is primarily attributed to the natural abundance of ^13^C. The peak at m/z =526.24 [A + 1] is 34% of the parent ion, indicating that the molecule contains 31 carbon atoms. When the reaction was performed in the sequence as for Fig. S6, where HmuF or FldH were added to the reaction mixture after HmuW converted deuteroheme to deuteroanaerobilin, then the mass of the dominant peak shifted by 4 and 6 units, yielding parent peaks at m/z = 529.28 [M + H]^+^ and m/z = 531.30 [M + H]^+^. Each of these parent peaks had an [A + 1] at 530.28 and 532.29. The MS data suggest that HmuF and FldH are able to catalyze multiple reductions of the linearized tetrapyrrole.

If HmuF is added at the beginning of the reaction, prior to the formation of deuteroanaerobilin by HmuW, then we observe primarily the 4-electron reduced form of anaerobilin as evidenced by a dominant parent peak at m/z = 529.28 [M + H]^+^ with a [A + 1] at 530.28, that is 34% of the parent ion (Fig. S8). Notably, only a minor peak corresponding to the 6-electron reduced form of anaerobilin is observed. These data indicate that coordination of heme to HmuF prior to HmuW catalysis limits the extent to which the tetrapyrrole is reduced.

These data also suggest that HmuF and HmuW form a protein–protein complex for radical-mediated decyclization of the heme and subsequent reduction of the tetrapyrrole. We looked for evidence of a protein–protein complex by monitoring shifts in the Soret maxima of Fe^2+^heme-loaded HmuF upon the addition of HmuW. As presented in Figure 3*A* and Fig. S9, Fe^2+^heme-loaded HmuF has Soret ƛ_max_ at 429 nm. The addition of an equimolar concentration of HmuW causes the Soret ƛ_max_ to shift to 425 nm, indicating a change in the coordination state of the iron (Fig. S9). Likewise, the addition of 10 μM HmuW to 10 μM Fe^2+^heme-loaded HmuF causes the Soret ƛ_max_ to shift from 405 to 408 nm. These small spectral shift in the Soret ƛ_max_ suggest formation of a HmuF–HmuW complex.

### Crystal structure of heme-loaded FldH

**Figure 8:**
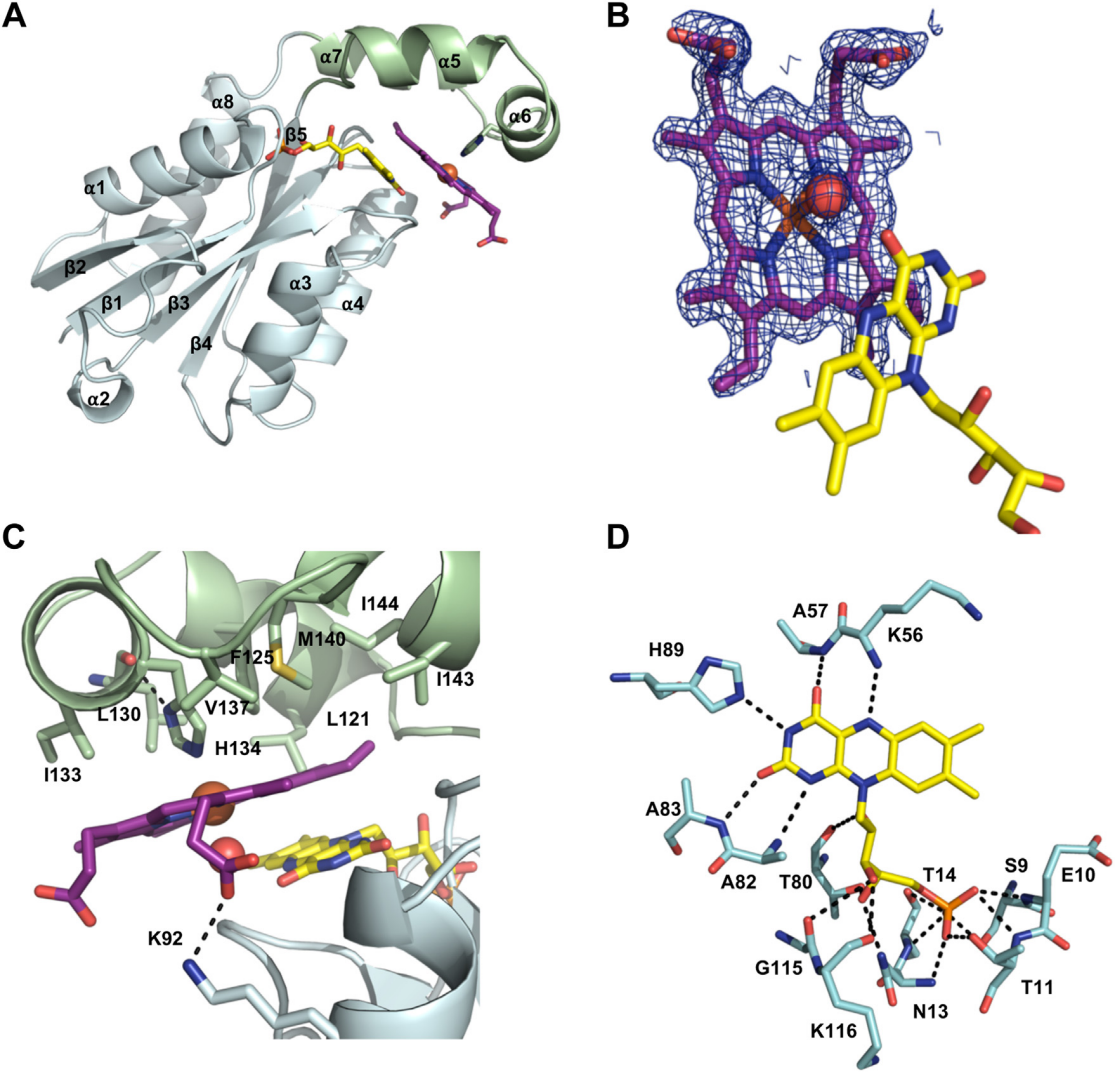
**Structure of Fe**^**3+**^**-heme FldH complex.***A*, cartoon structure of Fe^3+^-heme FldH with FMN and heme shown in *yellow* and *purple*, respectively. The flavodoxin fold is in *cyan* and the helical cap domain is colored *green*. *B*, a 2*F*_*o*_-*F*_*c*_ electron density map contoured at 1.5 σ for the heme. The orientation of the two cofactors shows the close proximity of N5 of the FMN and C5 of the heme macrocycle. *C*, close-up of the heme-binding site showing the clustering of hydrophobic residues that form van der Waals interactions with the proximal side of the tetrapyrrole ring. *D*, hydrogen-bonding interactions between FMN and protein.

Multiple attempts to form protein crystals of HmuF in the presence and absence of hemin were unsuccessful. However, brown crystals of heme-bound FldH were obtained. Phase information was obtained through iodide soaking experiments. The crystal structure was solved to a resolution of 1.6 Å (Table S2). All of the 169 residues of FldH are present in the structure along with the heme and FMN cofactors. A sulfate ion derived from the crystallization solution is also present. The single polypeptide present in the asymmetric unit adopts the typical flavodoxin-fold with five parallel β strands in a *β*2-*β*1-*β*3-*β*4-*β*8 arrangement flanked by five ⍺-helices (⍺1, ⍺2, ⍺3, ⍺4, ⍺8) (Fig. 8*A*). Size-exclusion chromatography combined with multi-angle light scattering indicates that the protein is monomeric, consistent with protein packing in the crystal structure. A notable feature of the structure is the helical cap domain (Lys116-His150) that bisects β5 and ⍺8 of the flavodoxin motif. This helical cap, comprising ⍺5, ⍺6, and ⍺7, provides a cleft for heme to be positioned planar to the *si*-face of the FMN isoalloxazine ring. Strong, unambiguous electron density is observed for the tetrapyrrole ring as shown in the 2*F*_o_ – *F*_c_ electron density map (Fig. 8*B*). The heme iron is coordinated on the proximal side by Nε of His134 in ⍺6 (Fe-Nε distance of 2.2 Å) and on the distal side by a water molecule, a coordination environment that is consistent with the electronic absorption spectrum of the heme-bound protein. N***δ*** of His134 forms a hydrogen bond with the carbonyl backbone of Leu130. The side chains of Leu121, Phe125, Leu130, Ile133, Val137, and Met140 form van der Waals interactions with the proximal side of the heme, creating a hydrophobic interface (Fig. 8*C*). An ion pair between the propionate group of the D pyrrole ring and the side chain of Lys92 (from ⍺3) further secures the heme to the protein. The orientation of the heme results in the B pyrrole ring forming pi-pi interactions with the pyrimidine ring of FMN. This places FMN N5, the site of hydride transfer, 3 Å from C5 of the tetrapyrrole ring (*i.e.*, at the ⍺-meso bridge). Intriguingly, C5 is the sp^2^-hybridized carbon proposed to be added by the methylene radical of the second SAM molecule during the RSMT reaction.

The FMN cofactor is situated at the carboxyl-terminal end of the β-parallel sheet, with the phosphate group secured through noncovalent interactions to the highly conversed phosphate-binding loop (Ser9 – Thr14) observed in other flavodoxin structures (Fig. 8*D*) (34). The ribityl interacts with the carbonyl groups of Thr80, Gly115, and Lys116 and the side chain of Asn13. The isoalloxazine ring hydrogen bonds with the side chain of His89 (to N3), the N backbone atoms of Lys56 (to N5), Ala57 (to O4), Ala82 (to N1), and N of Ala83 (to O2).

To determine which residues are conserved among putative heme-binding/FMN reductases, we performed a sequence alignment of HmuF homologs associated with heme uptake/utilization gene clusters. As presented in Fig. S10, His134 is present in the majority of homologs, with the exceptions being HmuF from several species of *Campylobacter* (*Campylobacter sputorum*, *Campylobacter geochelonis*, and *Campylobacter gracilis*) and *L. trevisanii* and *L. hofstadii*. Several residues in the phosphate-binding loop are conserved, as are residues that engage in hydrogen bond contact with the FMN isoalloxazine ring and ribityl. Residues Ala147, His150, Pro151, Asp155 along one of two linkers between the flavodoxin-like domain and the helical cap are also conserved.

## Discussion

Given that the vast majority of iron in the mammalian host is sequestered in heme proteins, the ability of bacterial pathogens to acquire heme is advantageous for host colonization. Once internalized, the heme can be enzymatically degraded to extricate iron or repurposed for other heme-dependent proteins. The balance between heme uptake and intracellular utilization needs to be tightly regulated as the accumulation of intracellular free heme is detrimental to bacterial growth: the porphyrin ring is lipophilic and the redox active iron can generate reactive oxygen species (35). Consequently, many heme uptake operons also encode heme-degrading enzymes, with heme oxygenases being the most well characterized (36). The heme uptake/utilization operons of *F. nucleatum* and the enteric pathogens *V. cholera* and *E. coli* O157:H7 encode an anaerobilin synthase that—unlike heme oxygenase—catalyze opening of the porphyrin ring and release of Fe^2+^ without the aid of molecular oxygen. However, the product of this reaction, anaerobilin is also cytotoxic, necessitating further enzymatic reduction.

Herein, we have shown that HmuF aids in heme utilization by trafficking intracellular heme to HmuW and catalyzing multiple reductions of anaerobilin. The structure of its paralog, heme-bound FldH provides insight into how the protein can fulfill both functions. First, the helical cap domain provides a hydrophobic binding interface for the porphyrin ring and His134 as the proximal iron ligand. Second, the heme is positioned over the FMN isoalloxazine ring to facilitate direct hydride transfer from the reduced FMN N(5)H to the tetrapyrrole. Based on the MS/MS fragmentation pattern of anaerobilin, LaMattina *et al.* proposed that the ⍺-meso bridge (*i.e.*, methine bridge involving C4-C5 or C5-C6) is broken during catalysis following transfer of a methyl group to the heme C5. β-scission leads to formation of a new vinyl group as shown in Figure 2. In the heme-bound FldH structure, C5 of the tetrapyrrole ring is ∼3 Å from FMN N5. We speculate that the FMN hydroquinone reduces this vinyl group, a reaction that likely lowers the cellular toxicity of anaerobilin. The newly installed methylene is a good Michael acceptor due to its conjugation with the pyrrole nitrogen. Reducing it to a methyl group eliminates its reactivity with cellular nucleophiles (*i.e.*, free thiols). Given that the ⍺-*meso* carbon is situated near the FMN, we speculate that interaction between HmuF and HmuW causes the helical cap and heme to rotate away from the FMN and flavodoxin motif, enabling the heme to serve as a substrate for HmuW. A shift in the Soret maxima of heme-loaded HmuF upon the addition of HmuW suggests that the two proteins form a complex. Once the HmuW RSMT reaction occurs and iron is released, the helical cap rotates towards the FMN for subsequent reduction of the newly installed vinyl group on anaerobilin.

**Figure 9:**
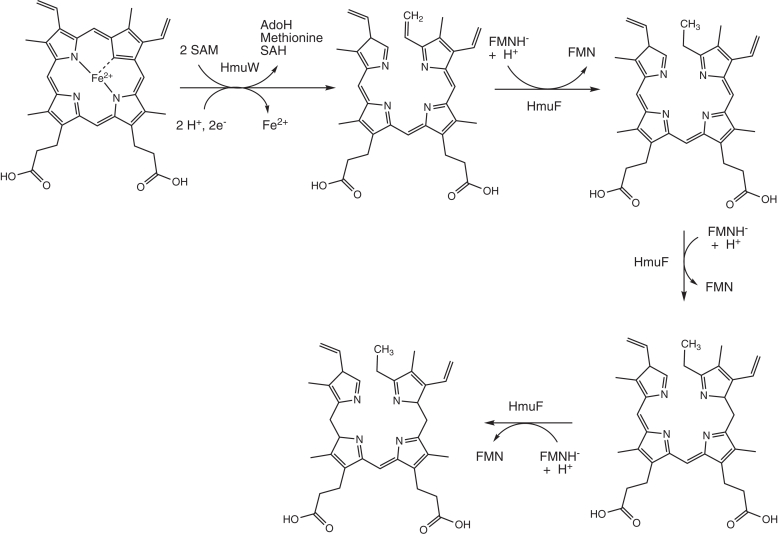
**Proposed mechanism for HmuF reduction of anaerobilin generated through HmuW turnover**.

If anaerobilin is first generated in the absence of HmuF, then the MS data reveal at most another 4-electron of the tetrapyrrole. These additional reductions may occur at two other meso-bridges, paralleling that of biliverdin reductase (Fig. 9). Multiple reductions would cause loss of conjugation along the tetrapyrrole ring, accounting for loss of absorbance at 445 and 795 nm when HmuF is added to anaerobilin in the presence of reducing agents. It is envisioned that anaerobilin adopts multiple conformations in the heme-binding cleft of HmuF to facilitate successive hydride transfer events. The hydrophobic properties of both the substrate and active site likely facilitate multiple conformations of the tetrapyrrole and hence multiple hydride transfers. Intriguingly, if heme is trafficked to HmuW for catalysis by HmuF, then a 4-electron–reduced anaerobilin product is generated, which suggests the prior coordination of HmuF leads to more control in the positioning of anaerobilin in the HmuF active site following the RMST reaction.

The heme uptake/utilization operons of *E. coli* O157:H7 and *V. cholera* also encode cytosolic heme-binding proteins, ChuX, ChuS, and HutX. All three proteins adopt similar folds (antiparallel eight stranded β-sheet and peripheral ⍺-helices) and bind Fe^3+^-heme with a 1:1 stoichiometry (37, 38, 39). In *E. coli* O157:H7, ChuS appears to function similarly to that of HmuF/FldH in that it traffics heme to ChuW for radical-mediated decyclization (25). Interestingly, the crystal structure of a heme-bound ChuS shows the ⍺-meso carbon of the heme exposed to the solvent, seemingly in position for radical-mediated methylation by ChuW (39). The importance of ChuS in maintaining heme homeostasis was established in *Shigella dysenteriae*, where disruption of *shuS*, which encodes a ChuS homolog, showed an impaired use of heme as an iron source at low heme concentrations and lethality at high heme concentrations (>40 μM) (40).

The *E. coli* O157:H7 heme uptake/utilization gene cluster also encodes ChuY, which catalyzes multiple reductions of anaerobilin using reducing equivalents from NADPH. ChuY reduces the β or ***δ***-meso positions of the porphyrin ring analogous to that of biliverdin IX β-reductase (24). In fact, the protein structure of ChuY is similar to that of biliverdin IX β-reductase (41). Deletion of ChuY from *E. coli* CFT073 lead to a reduced ability of the organism to infect embryonic kidney cells, which may be due to accumulation of heme or anaerobilin to toxic levels (42). Thus, while *E. coli* O157:H7 and *F. nucleatum* possess homologous RSMTs for opening of the porphyrin ring, they harbor distinct proteins for sequestering and trafficking heme and for mediating further reduction of the tetrapyrrole.

HmuF/FldH represent a new subclass of the flavodoxin-like superfamily that mediate hydride transfer reactions instead of single electron transfers. Other family members that mediate hydride transfer reactions include NADPH-dependent FMN reductases, WrbA-like proteins, and quinone reductase (43). Similar to that of FldH, these enzymes lack an aromatic residue shielding the *si*-face of the flavin isoalloxazine ring, a structural feature of flavodoxins. Exposure of the *si*-face ostensibly allows for hydride transfer reactions and may destabilize the semiquinone form of the cofactor. Other FMN reductases operate *via* a ping-pong mechanism whereby NAD(P)H donates hydride to the *si* face of the oxidized FMN cofactor. Following release of NADP^+^, a second FMN, quinone or other substrate binds to the same site and is reduced by the FMN hydroquinone. In crystal structures of two family members (SsuE/EmoB), a second FMN molecule (the hydride acceptor) occupies the same approximate binding site as the heme in the Fe^3+^-heme–FldH complex (43, 44). However, there are two notable differences between these FMN reductases and HmuF/FldH. First, NADPH does not directly donate a hydride to the FMN of HmuF or FldH. Instead, the HmuF/FldH FMN can be reduced through two consecutive single electron transfer events from NADPH-dependent flavin reductases. Second, the other reductases are dimeric or tetrameric, with the additional subunit contributing to binding of the second cofactor. HmuF/FldH are monomeric and possess an extra subdomain that secures the second cofactor, which is heme in this case.

Spectroscopic studies of HmuF and FldH established that binding of NO to Fe^2+^-heme ruptures the bond between His134 and Fe^2+^. Unlike CO or O_2_, NO exerts a negative *trans* effect that lengthens the Fe^2+^-His bond (45). In some protein scaffolds (*e.g.*, soluble guanylate cyclase, FixL, and cytochrome c’), this lengthening induced breakage of the Fe^2+^-His bond. Other proteins, such as myoglobin and horse radish peroxidase, remain 6-coordinate with NO bound (44, 46). It has been suggested that weak Fe^2+^-His bonds rupture upon NO binding. The Fe^2+^-His bond strength depends to some degree on the capacity of the imidazole side chain to donate negative charge to the heme Fe^2+^, which is correlated to the hydrogen-bonding capacity of His N***δ***. In the crystal structure of FldH, N***δ*** of His143 engages in hydrogen bond contact with a carbonyl group, which should increase its σ-donation capacity to Fe^2+^. However, QM/MM calculations with cytochrome c’ also suggest that rigidity of the protein matrix influences whether the histidine residue dissociates upon NO binding (47). His134 is a part of the helical cap domain, which is likely a mobile element given that it needs to rotate in order to facilitate catalysis by HmuW. We conjecture that the flexibility of the helical cap domain enables breakage of the Fe^2+^-His bond in the presence of NO. It is not known if binding of NO (or CO) is biologically significant, but it is worth noting the K_D_ values for NO–heme protein complexes range between 10^−7^ to 10^−10^ M (48), a concentration range that overlaps with that of cellular NO concentrations (5 × 10^−9^ M to 10^−10^) (49).

In summary, we have presented the structure and function of a new member of the flavodoxin superfamily that participates in the anaerobic catabolism of heme. By sequestering and trafficking heme to HmuW, HmuF protects the cell from the cytotoxic effects of labile heme. HmuF further safeguards the cell from the product of HmuW turnover by catalyzing multiple reductions of the linearized tetrapyrrole. Through its dual function, HmuF supplants the role of ChuX/ChuS and ChuY in the anaerobic heme degradation in *E. coli* O157:H7. The combined function of HmuF may provide *F. nucleatum* with a competitive advantage over other microorganisms in anoxic environments such as the subgingival plaque or the colon. The distinct structure of HmuF also provides a new platform for the design of antimicrobials to compact *F. nucleatum* infections.

## Experimental procedures

### Plasmid construction

The genes corresponding to FldH (FN1822), HmuF (FN0772), and HmuW (FN0771) were amplified from the genomic DNA of *F. nucleatum* subsp. *nucleatum* ATCC 25586 using Q5 DNA polymerase. The forward primers harbored an *Nde*I restriction site, while the reverse primers contained either *Bam*HI or *Hind*III restriction sites (Table S3). The PCR products corresponding to FldH and HmuF were subcloned into the *Nde*I-*Bam*HI site of pET15b, while the DNA fragments harboring *hmuW* were ligated into the *Nde*I-*Hind*III site of pET26b, respectively, producing constructs for hexahistidine-tagged versions of HmuF (N-terminal), FldH (N-terminal), and HmuW (C-terminal). The resulting constructs were sequenced to confirm the absence of PCR-induced errors.

### Expression and purification of FldH and HmuF

The *E. coli* strain BL21(DE3)pLysS was transformed with pET15_HmuF or pET15_FldH, and a single transformed colony was used to inoculate 100 ml of LB medium supplemented with 100 μg/ml of ampicillin and 30 μg/ml of chloramphenicol. The culture was grown overnight at 37 °C at 200 rpm, and then 10 ml of this starter culture was used to inoculate 1 l of 2 YT also supplemented with 100 μg/ml of ampicillin and 30 μg/ml of chloramphenicol. The culture was incubated at 30 °C with shaking at 200 rpm. At an A_600_ of 0.6 to 0.8, 0.2 mM of IPTG was added to the culture to induce transcription. The cultures continued to grow at 25 °C at 200 rpm for an additional 16 h. The cells were harvested by centrifugation, and the cell pellet was stored at −70 °C until protein purification.

To purify FldH and HmuF, ∼20 g of cell pellet was resuspended in 100 ml of 50 mM Tris–HCl, pH 7.5, 0.15 M NaCl (Buffer A) with 0.5 mM of PMSF. The cell suspension was sonicated for 30 min (30 s pulses at 1 min intervals) and then clarified by centrifugation at 38,000*g* for 60 min. Imidazole was added to the supernatant to achieve a final concentration of 10 mM. The crude extract was then applied to 5 ml fast flow Ni-NTA column equilibrated with buffer A containing 10 mM imidazole. The column was washed with 50 ml of buffer A with 10 mM imidazole and then the proteins were eluted with buffer A containing 250 mM imidazole. The amber colored protein fractions were pooled and then dialyzed against 50 mM Tris–HCl for 18 h at 4 °C in the presence of thrombin to cleave the hexahistidine tag. The dialysate was re-applied to a Ni-NTA acid column equilibrated with buffer A with 10 mM imidazole to separate the cleaved protein fraction. The flow through from the fraction was applied to a Q Sepharose column (2.6 × 14 cm) equilibrated with 50 mM Tris–HCl, pH 7.4. The column was washed with two column volumes of 50 mM Tris–HCl, pH 8.0 prior to running a linear gradient from 0 to 0.5 M NaCl. Both FldH and HmuF eluted in the early part of the gradient. The purity of the protein was verified by 12% SDS-PAGE and Coomassie blue staining.

### Expression and purification of HmuW

The expression construct for HmuW, pET26b_HmuW (kanamycin resistance), was cotransformed with pDB1282 (ampicillin resistance) into *E. coli* strain BL21(DE3). The plasmid pDB1282 (a kind gift from Dr Dennis Dean at Virginia Tech) is often employed for the soluble expression of radical SAM enzymes as it harbors the *isc* operon from *Azotobacter vinelandii*, which encodes machinery for the biosynthesis and maturation of Fe/S centers (50). Co-expression of radical SAM enzymes in the presence of the *isc* operon has been shown to improve their solubility, likely due to enhanced Fe/S maturation (50). Individual transformed colonies were used to inoculate 100 ml of LB medium supplemented with 100 μg/ml of ampicillin and 50 μg/ml of kanamycin. The starter cultures grew at 37 °C for 16 h at 200 rpms. Fifteen milliliters of the starter culture was used to inoculate 1.5 l of M9 minimal medium in 2 l culture flasks prewarmed to 37 °C. Ampicillin (100 μg/ml), kanamycin (50 μg/ml), and FeCl_3_ (25 μM) were added to the culture media at the time of inoculation. The cultures were incubated at 37 °C with shaking at 180 rpm until an A_600_ of 0.3, at which time L-(+)-arabinose, L-cysteine, and FeCl_3_ were added to the media to final concentrations of 0.2% (w/v), 300 μM and 25 μM, respectively. The cultures continued to grow at 37 °C until an A_600_ of 0.6 at which time they were briefly chilled in an ice water bath, and IPTG was added to the cultures to a final concentration of 0.2 mM. The cells continued to grow at 18 °C at 180 rpms for an additional 18 h. Cells were harvested by centrifugation (10,000*g* at 4 °C for 10 min) and stored at −70 °C until purification.

### Purification of His-tagged HmuW

HmuW was purified in a glove box (Belle Technology) maintained under nitrogen atmosphere with O_2_ <5 ppm. Buffers used in the purification were extensively purged with 99.998% N_2_ prior to introduction to the glove box, where they were allowed to equilibrate for >16 h prior to use. Buffers were chilled in an ice bath before use. The cell pellet (∼15 g) was introduced into the glove box and resuspended on ice with 100 ml of chilled buffer A (20 mM Hepes, pH 7.8, 300 mM NaCl) supplemented with 0.1 mM PMSF and 2 mM imidazole. The cells were sonicated on ice for 30 min (15 s pulses with 1 min intervals). The lysate was centrifuged for 1 h at 4 °C at 50,000*g*. The clarified extract was applied to a gravity flow Talon cobalt resin (2.5 cm × 10 cm) equilibrated with buffer A containing 2 mM imidazole. The column was washed with 50 ml of buffer A containing 2 mM imidazole. The protein was eluted with buffer A with 10% glycerol (v/v) and 100 mM imidazole.

The Fe/S clusters were incorporated into HmuW by following a published protocol (50). In the anaerobically maintained glove box, HmuW was diluted to 70 μM in buffer A with 10% glycerol and 10 mM DTT. After 30 min, 0.2 mM of ferrous ammonium sulfate [Fe(NH_4_)_2_(SO_4_)_2_] was added to the protein solution. Following a 30 min incubation, 15 μl of 100 mM sodium sulfide (Na_2_S 9H_2_O) was added to the protein solution every 10 min until a final concentration of 0.2 mM was achieved. The mixture was then stored at 4 °C for 16 h. The protein was concentrated using an Amicon stirred cell with a 30 kDa cut-off filter. A 3D-printed plastic “moat” filled with ice was placed around the stirred cell to keep the protein from precipitating during concentration. Excess iron and sulfide were removed by repeated dilutions of the protein with Buffer A with 10% glycerol followed by concentration in an Amicon stirred cell. The brownish protein was used immediately for enzymatic assays. Purity was assessed visually by the analysis of Coomassie-stained SDS-PAGE gel. Protein concentrations were determined using absorbance at 280 nm using a theoretical extinction coefficient calculated by the ExPASy ProtParam tool.

### Iron quantification

The ferrozine assay was used to quantitate the amount of iron in HmuW after reconstitution (51). A solution containing 300 μl of HmuW, 333 μl of 2 M guanidine hydrochloride, 40 μl of 12 M HCl, and 100 μl of 100 mM freshly prepared L-ascorbic acid was mixed thoroughly. The solution was centrifuged to remove the insoluble particulates. The supernatant was neutralized with 100 μl of 5 M ammonium acetate and then 10 μl of 100 mM ferrozine (3-(2-pridyl)-5,6-diphenyl-1,2,4-triazine-p,p’-disulfonic acid monosodium salt hydrate) was added. The solution was diluted to 1 ml and then incubated at 21 °C for 30 min. The absorbance of the iron–ferrozine complex was recorded at 562 nm using a Lambda 25 UV-visible spectrophotometer (PerkinElmer). The iron content was determined by comparing this reading to a standard curve that was generated under identical conditions using ferrous ammonium sulfate (Fe(NH_4_)_2_(SO_4_)_2_) with a concentration range from 0 to 120 μM.

### Sulfide quantification

The amount of sulfide incorporated into HmuW was determined through an assay involving methylene blue (52). One ml of 1% (w/v) zinc acetate was added to 300 μl HmuW followed by 50 μl of 3 M NaOH. Following gentle agitation of the solution, 250 μl of 0.1% N, N-dimethyl-p-phenylenediamine monohydrochloride in 5 M HCl and 50 μl of 23 mM FeCl_3_ in 1.2 M HCl were added simultaneously. The resulting solution was mixed vigorously for 5 min intervals for a total of 30 min. The samples were then centrifuged at 16,000*g* for 5 min at 25 °C. The supernatant was collected, and the absorbance at 670 nm was recorded using a Lambda 25 spectrophotometer. The sulfide content was determined by comparing the reading to a standard curve generated under identical conditions using a fresh solution of sodium sulfide (Na_2_S) in 0.1 M NaOH with a concentration range of 0 to 100 μM.

### Purification of the FAD-NADPH reductase domain from *A. annua* cytochrome P450 reductase (aaCPR_FAD_) and FnFld (FN0724)

For enzymatic assays involving HmuW, we used aaCPR_FAD_ as an electron conduit between NADPH and FnFld. The FAD/NADP(H) domain is homologous to flavodoxin NADPH reductase from *E. coli* and can transfer electrons from NADPH to a variety of flavodoxins. The N-terminal–expressed aaCPR_FAD_ was expressed and purified as previously described (53). Our group previously characterized a flavodoxin from *F. nucleatum* (FnFld; FN0724), which was also used in kinetic assays with HmuW. FnFld was expressed and purified as described previously (33).

### Potentiometric titrations

Redox titrations of HmuF and heme-bound HmuF and FldH were performed in the anaerobically maintained glove box. The titration buffer, 50 mM Hepes–NaOH pH 7.0 with 10% (v/v) glycerol was made anaerobic by bubbling it with N_2_ for 2 h followed by overnight equilibration in the glove box. The hemin stock solution was freshly prepared by as described for the heme-binding assays. For titrations of heme-bound HmuF and FldH, each protein was reconstituted with an equimolar amount of hemin. The heme-loaded protein was introduced to the glove box and then filtered over a 10 ml PD10 column (Bio-Rad) equilibrated with the anaerobic titration buffer. For redox titrations of HmuF (devoid of heme), a 2 ml solution of flavoprotein was introduced into the glove box exchanged into the titration buffer using a PD10 column. The heme-containing proteins were diluted using the anaerobic titration buffer to around 10 μM in a total volume of 10 ml while HmuF was diluted to ∼50 μM. Redox mediators, 1 to 2 μM methyl viologen (*E*_m_= −450 mV), benzyl viologen (*E*_m_ = −358 mV), phenazine methosulfate (*E*_m_ = 80 mV), and 2-hydroxy-1-4-napthoquinone (*E*_m_ = −137 mV), were added to the protein solution to mediate between the range of +100 mV and −480 mV. The oxidized protein was gradually reduced with the addition of 1 μl aliquots of sodium dithionite. The absorbance spectra were recorded with a Lambda 265 UV/visible spectrophotometer (PerkinElmer), and the potential was measured using a pH/ORP meter (Orion 3-Star Benchtop Meter, Thermo Fisher Scientific) equipped with an ORP electrode (Orion 9179 BNMD, Thermo Fisher Scientific). Both instruments were housed in the glove box. After each addition of dithionite, the potential was recorded once the protein solution had equilibrated and the absorbance spectrum was recorded. Plots of the absorbance against redox potential were fitted to Equation 3, which is derived by extension of the Nernst equation and the Beer-Lambert Law as described previously (54).

$$A_{obs}=\frac{\left(a+{b10}^{\left(E_{12}-E\right)/59}\right)}{1+10^{\left(E_{12}-E\right)/59}}$$

In equation 1, A_obs_ is the absorbance value of Fe^3+^-heme or the oxidized FMN cofactor, E is the electrode potential, a and b are the absorbance values of the fully oxidized and reduced cofactor at a specified wavelength, and E_12_ is the midpoint potential for the concerted two-electron reduction of the FMN or one-electron reduction of Fe^3+^-heme. Data manipulation and analysis were performed using Origin software package version 8.0 (https://www.originlab.com/). All redox potentials are given relative to the normal hydrogen electrode.

### Monitoring HmuW activity by UV-visible spectroscopy

UV-visible absorbance spectra were recorded on a PerkinElmer Lambda 265 diode array spectrophotometer housed in the anaerobic glove box. Solutions of FnFld and aaCPR_FAD_ were made free of O_2_ by introducing concentrated stocks of each protein into the glove box and buffer exchanging them into anaerobic buffer using a PD10 column. Solutions of NADPH and SAM were prepared by dissolving the powdered forms of the substrates in the glove box with anaerobic buffer. An anaerobic solution of hemin chloride was also made in the glove box with 0.1 M NaOH free of O_2_. Spectral analysis of HmuW-mediated formation of anaerobilin was conducted in 50 mM Hepes–NaOH pH 7.0 containing 5% v/v DMSO, 2 μM HmuW, 2 μM FnFld, 2 μM aaCPR_FAD_, 250 μM NADPH, and 10 μM of hemin. The reactions were initiated with the addition of SAM to a final concentration of 250 μM. Spectra were acquired from 350 to 900 nm every 2 min.

### MS of reaction products

MS was used to determine if HmuF lead to reduction of anaerobilin. Due to the limited solubility of heme, the reaction was performed with deuteroheme. Three 5 ml reactions containing 20 μM deuteroheme, 10 μM HmuW, 400 μM SAM, 500 μM NADPH, 20 μM FnFld, and 5 μM aaCPR_FAD_ were prepared in 50 mM Hepes–NaOH, pH 7.5, 0.3 M KCl, and 20% v/v glycerol. Following a 2-h incubation in the dark, the UV-visible spectra of the reaction mixture was recorded to confirm complete conversion to deuteroanaerobilin. Once the reaction was complete, 10 μM HmuF was added to one vial, while 10 μM FldH was added to a second reaction. The reactions were incubated in the dark for 15 min. A separate (fourth) 5 ml reaction was also prepared in which HmuF was added at the beginning of the reaction. This 5 ml reaction contained 30 μM deuteroheme, 10 μM HmuW, 500 μM NADPH, 20 μM FnFld, 5 μM HmuF, and 5 μM aaCPR_FAD_ and was initiated with the addition of 400 μM SAM. The reaction proceeded for 4 h in the dark at 20 °C. As described in LaMattina *et al.*, each 5 ml reaction was then applied to a C1 sep pack column (Silicycle) equilibrated in succession with 1 eq of methanol, 1 eq of dH_2_O, and 1 eq of assay buffer. Following application of the assay mixture to the columns, the columns were then washed with 1 eq of assay buffer, 2 eq of dH_2_O, 1 eq of 1% TFA, and 3 eq of dH_2_O. The linearized tetrapyrroles were eluted with 1 eq of 100% methanol. The eluent was analyzed by electrospray ionization by direct infusion into a Thermo Scientific Q Exactive Orbitrap Mass Spectrometer with the capillary temperature set to 220 °C and a syringe follow rate of 15 μl/min. Data were acquired over 1 minute and then averaged.

### X-ray crystallography

Purified FldH was concentrated to 25 mg/ml and mixed with an equimolar solution of hemin chloride prepared in 0.1 M NaOH and 10% (v/v) DMSO. The protein solution was applied to a PD10 desalting column to remove unbound heme and then screened using the JCSG Core Screen set. Crystals were obtained using the sitting-drop vapor diffusion method by mixing 1 μl of 16 mg/ml hemin-FldH solution (in 20 mM Tris, pH 8.0) and 1 μl of reservoir solution consisting of 0.1 M phosphate/citrate pH 4.2, 2.0 M NH_4_SO_4_. A crystal was then soaked for 1 h in 0.4 M NaI, 20% glycerol prepared in well solution, and five anomalous diffraction datasets were collected at the UBC ASTRID homesource facility. These datasets were processed to 1.95 Å in space group P321 and scaled together using HKL3000 (55). The data were then reindexed to P3_1_21, and the iodide-containing structure was successfully determined using Phenix AutoSol (56). Iodide-free crystals produced in 0.1 M sodium citrate pH 5.5, 2 M NH_4_SO_4_ and soaked in 20% glycerol (v/v) prepared in well solution were then flash frozen and sent to the Canadian Light Source beamline CMCF-BM. A 1.6 Å dataset was collected and the structure was solved using the iodide-bound model as a search model for molecular replacement in Phenix PhaserMR, followed by manual building in WinCoot (57). The atomic coordinates and structure factors of heme-bound FldH have been deposited in the Protein Data Bank under PDB ID 8G64.

## Data availability

Protein coordinates and structure factors have been submitted to the Protein Data Bank under accession code 8G64.

## Supporting information

This article contains supporting information.

## Conflict of interest

The authors declare that they have no conflicts of interest with the contents of this article.
