# Supplementary material for: A new member of the flavodoxin superfamily from Fusobacterium nucleatum that functions in heme trafficking and reduction of anaerobilin
Source: J Biol Chem. 2023 Jun 10;299(7):104902. doi: 10.1016/j.jbc.2023.104902 (PMC10404700; doi:10.1016/j.jbc.2023.104902)
Supplement: Supporting Figures S1–S10 and Tables S1–S3 [file mmc1.docx]

**Supporting Information**

**A new member of the flavodoxin-superfamily from *Fusobacterium nucleatum* that functions in heme-trafficking and reduction of anaerobilin**

Alexandra K. McGregor,^1^ Anson C.K. Chan,^2^ Megan D. Schroeder,^1^ Long T.M. Do^1^, Gurpreet Saini, Michael E.P. Murphy,^2^ Kirsten R. Wolthers^1^*

^1^Department of Chemistry, University of British Columbia, Okanagan campus, 3247 University Way, Kelowna, Canada

^2^Department of Microbiology and Immunology, Life Sciences Institute, University of British Columbia, Vancouver, Canada

**Table S1**. **Absorption maxima of heme-loaded HmuF and FldH in ferric and ferrous states and bound to CO and NO**. The extinction coefficient of the Soret band is shown in parenthesis (units of mM^-1^ cm^-1^).

| Protein | Soret max (nm) | ⍺/β (nm) | |
| --- | --- | --- | --- |
| FldH - Fe^3+^ | 405 (108.7) | 574 | 630 |
| FldH - Fe^2+^ | 424 (110.1) | 559 | |
| FldH - Fe^2+^-NO | 401 (71.0) | 535 | 565 |
| FldH - Fe^2+^-CO | 424 (134.5) | 539 | 568 |
| HmuF - Fe^3+^ | 405 (118.6) | 629 | |
| HmuF - Fe^2+^ | 429 (107.0) | 558 | |
| HmuF - Fe^2+^-NO | 401 (58.8) | 535 | |
| HmuF - Fe^2+^-CO | 421 (122.9) | 538 | 563 |

**Table S2: Data Collection and Refinement Statistics for Fe^3+^ heme-bound FldH**

| **Data Collection** |  |
| --- | --- |
| Resolution range (Å) | 37.86-1.60 (1.65-1.60) |
| Space group | *P* 3_1_21 |
| Unit cell dimensions (Å) | *a* = 67.24, *b* = 67.24, *c =* 99.65 |
| Wavelength | 0.99184 |
| Total reflections | 343047 |
| Unique reflections | 66288 |
| Completeness (%) | 99.6 (99.1) |
| Redundancy | 9.8 (9.9) |
| Average *I*/σ (*I*) | 18.49 (2.85) |
| *R*_merge_ | 0.067 (0.850) |
| CC_1/2_ | 0.999 (0.843) |
| **Refinement** |  |
| *R*_work_/*R*_free_ | 0.1596/0.1783 |
| No. protein atoms | 2768 |
| No. heme atoms | 73 |
| No. of FMN atoms | 49 |
| No. of waters | 264 |
| Average *B*-factors (Å^2^) | 29.64 |
| R.m.s.d bond lengths (Å) | 0.009 |
| R.m.s.d bond angles (°) | 0.862 |
| Ramachandran plot |  |
| Favoured (%) | 98.20 |
| Allowed (%) | 1.80 |
| Disallowed (%) | 0 |

** highest resolution shell in brackets*

**Table S3: Oligonucleotides used in this study**. F indicates forward primer while R indicates reverse primer. All sequences are provided 5ʹ to 3ʹ.

| Primer Name | Sequence |
| --- | --- |
| F_FN0772_NdeI | GGAATTCCATATGAAAACATTAATAGTTTATTCCAC |
| R_FN0772_BamHI | CGGGATCCTTAATTATTTAATAGATTAG |
| F_FN1822_NdeI | GGAATTCCATATGAAAACTTTAATAATTTATTCATC |
| R_Fn1822_BamHI | CGGGATCCTCAATAATTGAAATTTTTGTC |
| F_FN0771_NdeI | GGAATTCCATATGTTTAAAATAAGATATAAATCACATCATGATGTAG |
| R_FN0771_HindIII | GATAAGCTTTAAATGCCTCCCAATCTCTTCAATAATATCTG |


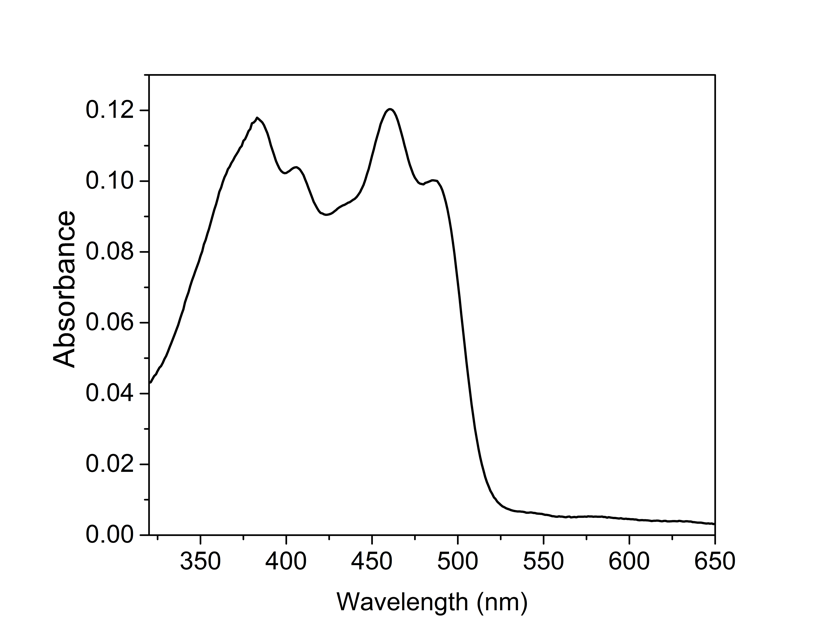


**Figure S1.** Absorbance spectrum of FldH obtained following elution from the Q-sepharose column.


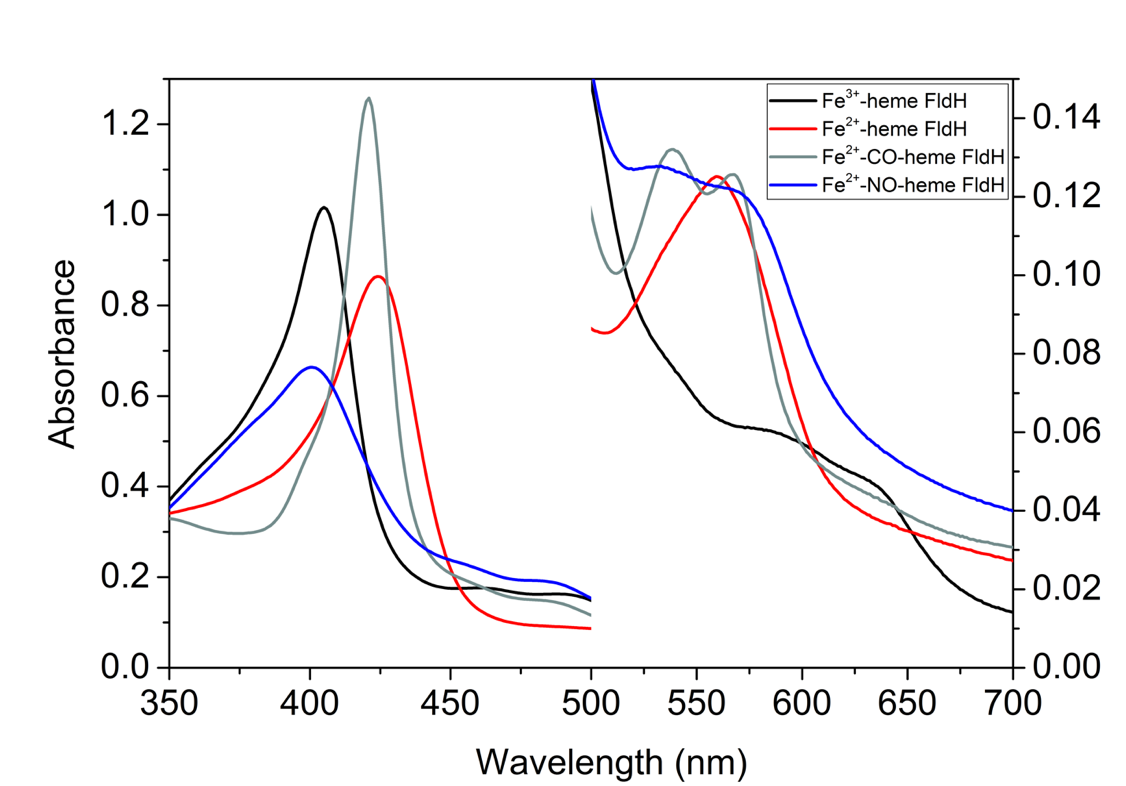


**Figure S2**. Heme-bound FldH in different ligation and redox state (black line, Fe^3+^-heme; grey line Fe^2+^ -heme; blue line NO-Fe^2+^ heme, and red line CO-Fe^2+^ heme).

**
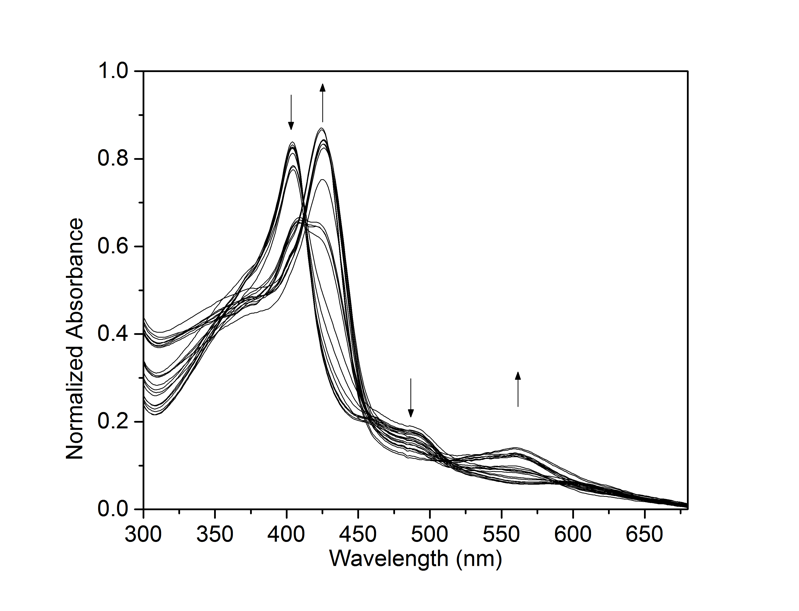

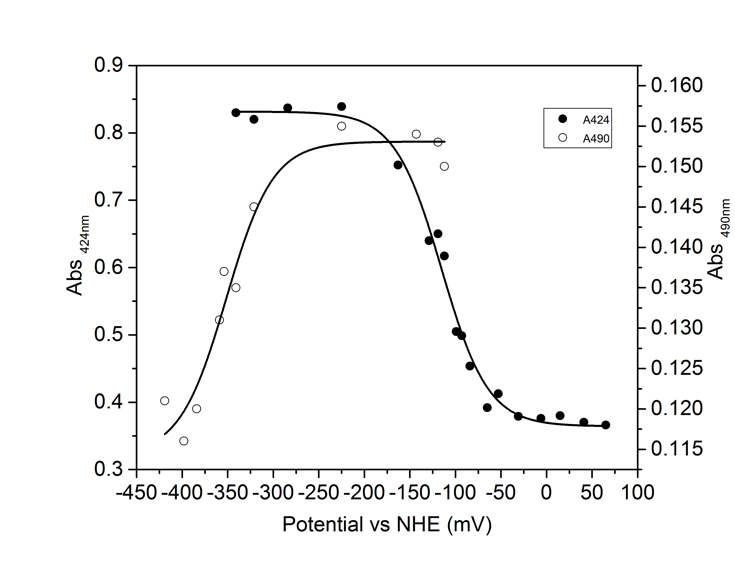
**

B

A

**Figure S3*. Chemical redox titrations of FldH.*** Absorption spectra for the redox titration of the heme-loaded HmuF. Electronic absorption spectra of (A) heme-bound FldH during a chemical reduction with dithionite. Arrows indicate direction of absorbance change. (B) Plot of absorbance against the reduction potential versus the normal hydrogen electrode. The open circles correspond to absorbance data at 490 nm (tracking reduction of the FMN cofactor) and the closed circles correspond to absorbance data at 424 nm (tracking formation of the Fe^2+^ heme). Data in panels B were fitted to eq 3 and the midpoint reduction potential for the Fe^3+^ /Fe^2+^ heme couple was -115 ± 5 mV and the FMN_ox/hq_ (oxidized/hydroquinone) couple was -352 ± 11 mV.


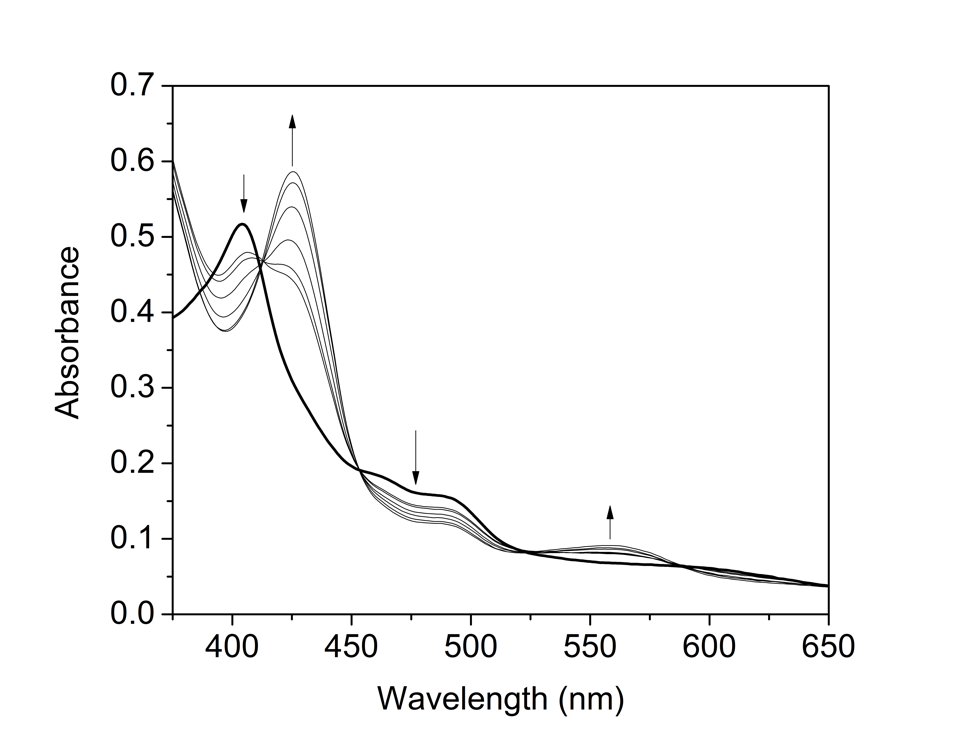


**Figure S4. aaCPR_FAD_ reduction of heme-bound FldH**. Electronic absorption spectra of 10 µM heme-bound FldH following the addition of 1 µM aaCPR_FAD_ and 250 µM NADPH. The black line is the spectrum of the protein prior to the addition of NADPH while grey lines are the spectra acquired every 30 s following the addition of NADPH. The arrows indicate the direction of the absorbance change.


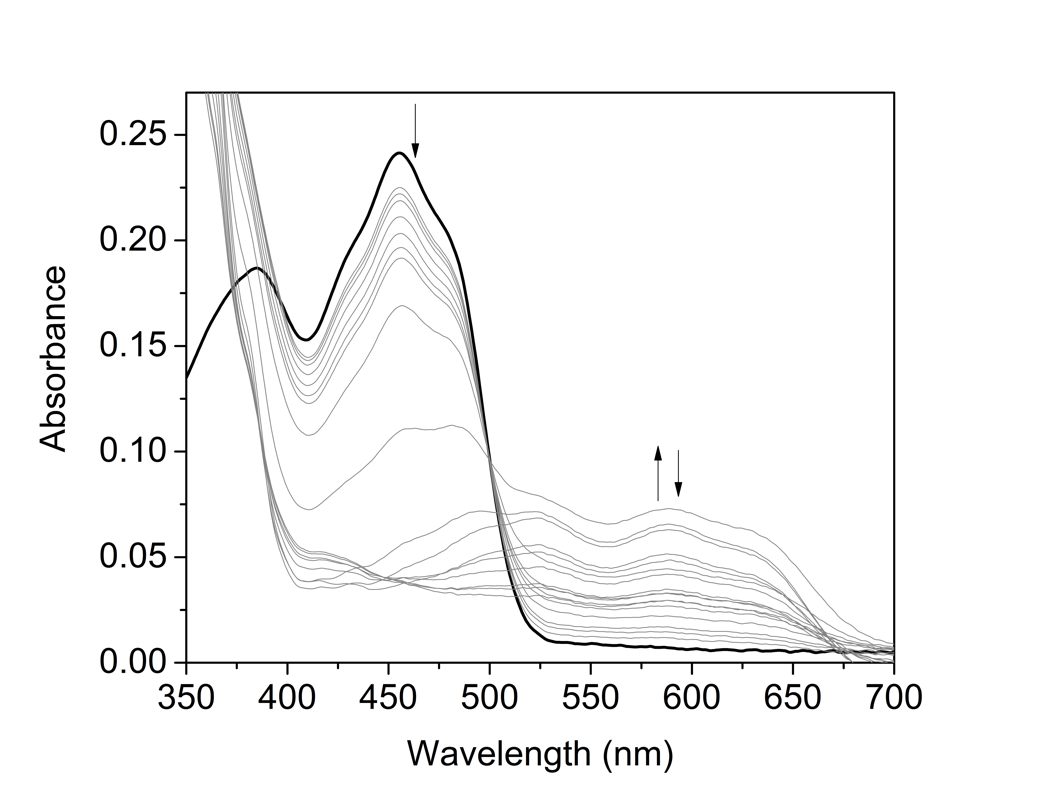


**Figure S5. NADPH-dependent reduction of FnFld by aaCPR.** Electronic absorption spectra of 18 µM FnFld following the addition of 250 µM NADPH and 0.5 µM aaCPR_FAD_ in 50 mM HEPES-NaOH, pH 7.8. The thick black line is the spectrum of FnFld before addition of NADPH and the grey lines were taken at various time points after addition. Complete reduction of FnFld took ~2 mins.


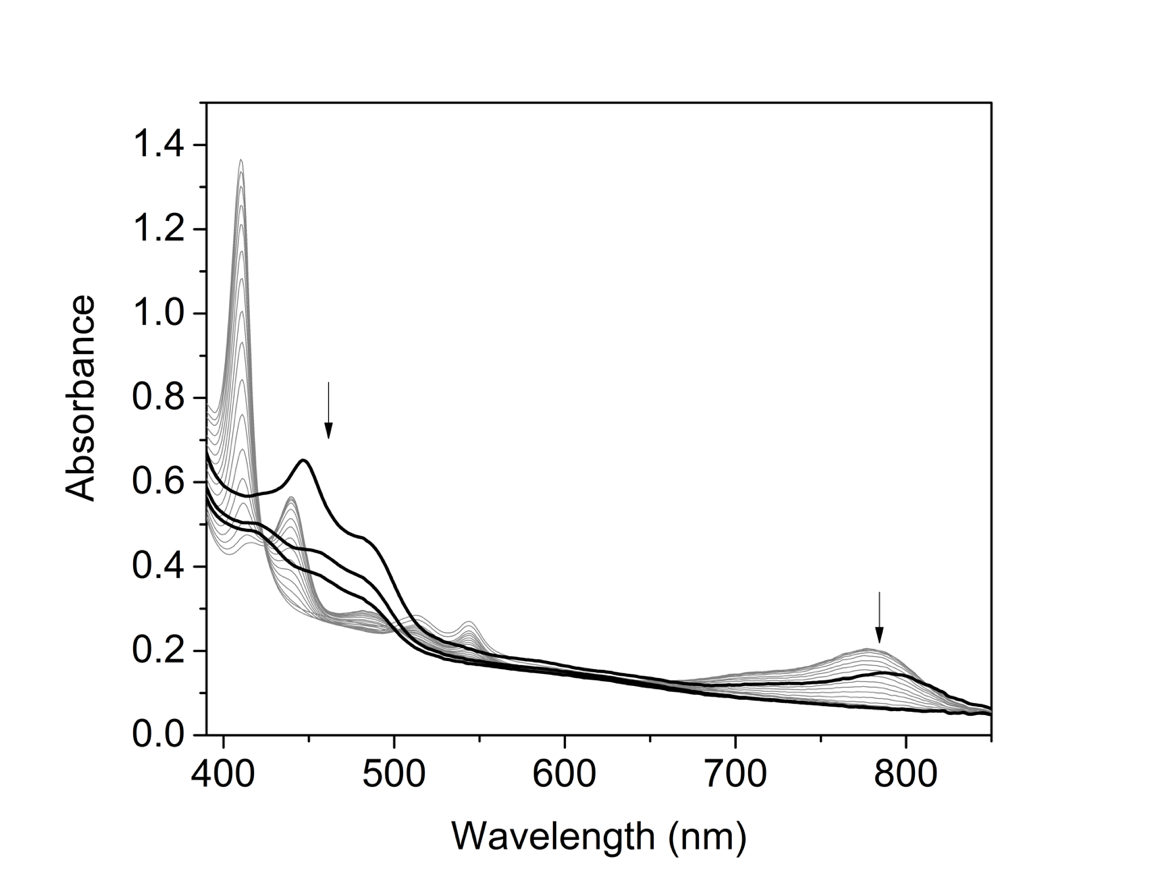


10 min

5 min

30 s

**Figure S6. Deuteroanaerobilin reduction by HmuF.** UV-visible spectroscopic assays were performed by adding HmuW (2 μM) to a solution containing 10 μM deuteroheme, 2 μM aaCPR_FAD_, 5 μM FnFld, 200 μM NADPH. The reaction was initiated with the addition of 250 μM SAM. The spectra were acquired every 2 min for 45 min (grey lines). The arrows indicate the direction of the absorbance change. Once anaerobilin was formed, denoted by no further absorbance changes at 795 nm, HmuF (10 μM) was added to the reaction. The black lines denote absorbance changes in anaerobilin at various time points.

**
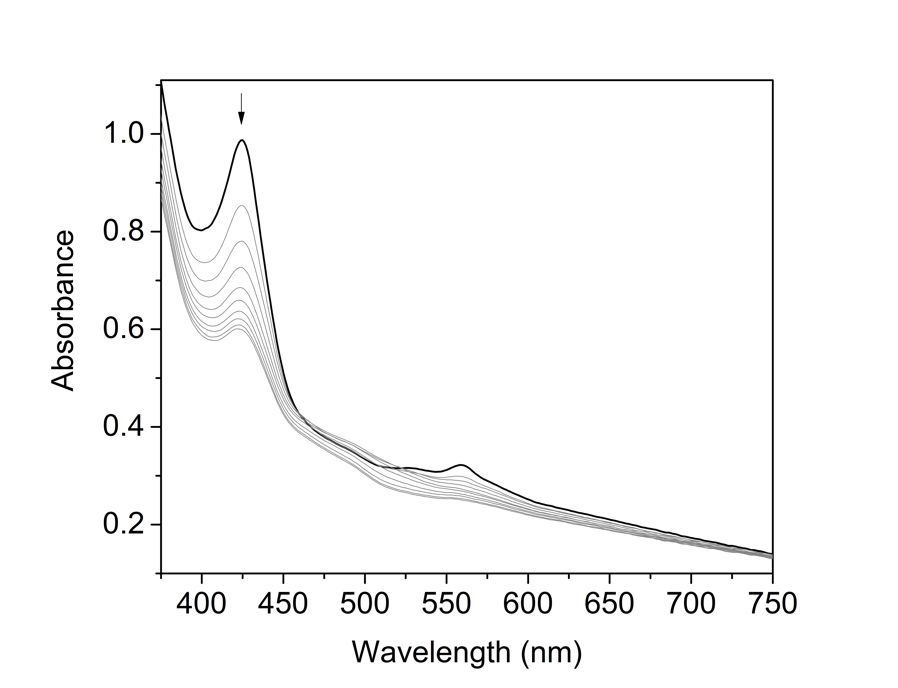
**
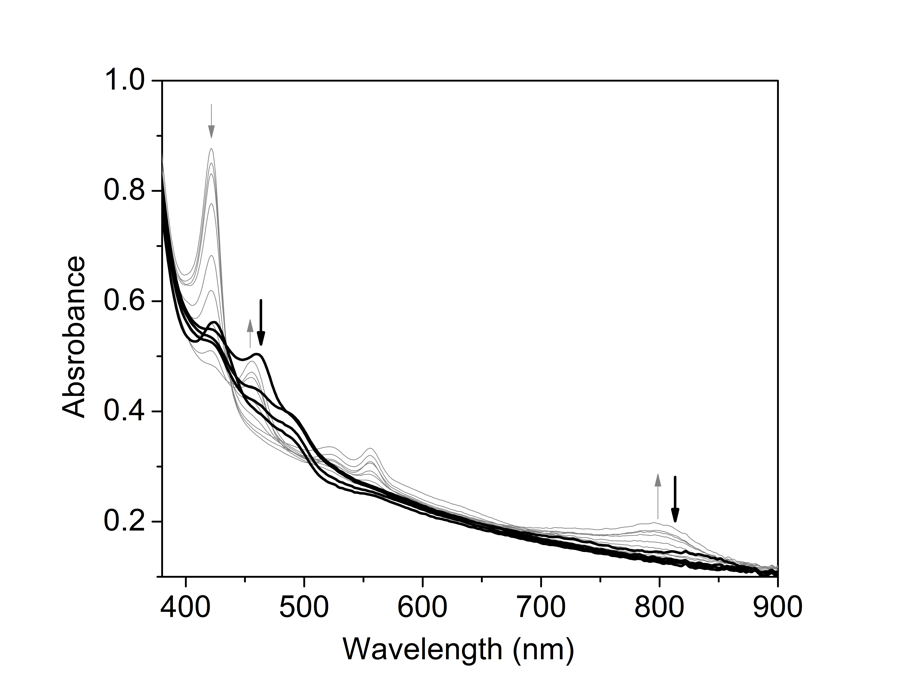


15 min

B

A

10 min

5 min

30 s

**Figure S7. Heme trafficking and anaerobilin reduction by FldH.** UV-visible spectroscopic assays were performed by adding HmuW (2 μM) to a solution containing (A) 10 μM hemin, 2 μM aaCPR_FAD_, 5 μM FnFld, 200 μM NADPH. The reaction was initiated with the addition of 250 μM SAM. The spectra were acquired every 2 min for 45 min (grey lines). The arrows indicate the direction of the absorbance change. Once anaerobilin was formed, denoted by no further absorbance changes at 795 nm, FldH (10 μM) was added to the reaction. The black lines denote absorbance changes in anaerobilin at various time points. (B) Absorbance change in 10 μM Fe^2+^-heme bound FldH following addition of 2 μM aaCPR_FAD_, 5 μM FnFld, 200 μM NADPH (black line). Absorbance changes in the heme Soret band following subsequent addition of HmuW (2 μM) and 250 μM SAM (grey lines). Spectra were acquired every 5 min for 40 min.

**
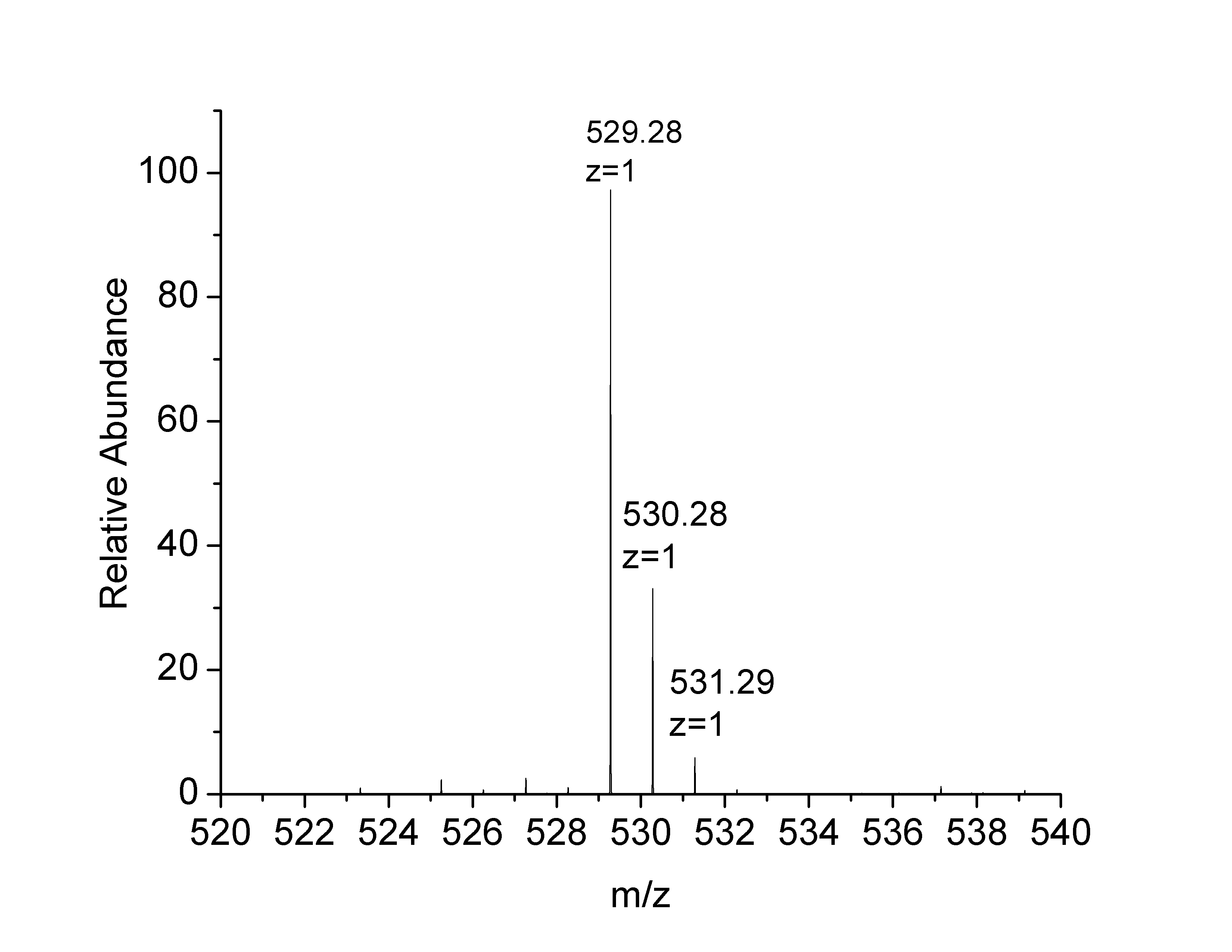
**

H^+^

**Figure S8**. Reduced deuteroanaerobilin products generated in a 5 mL reaction containing 10 μM HmuW, 400 μM SAM, 5 μM aaCPR, 20 μM FnFld, 500 μM NADPH, 30 μM deuteroheme, 5 μM HmuF in 50 mM Tris-HCl, pH 7.5, 0.3 M KCl and 20 % v/v glycerol. The reaction was initiated with the addition of 400 μM SAM.


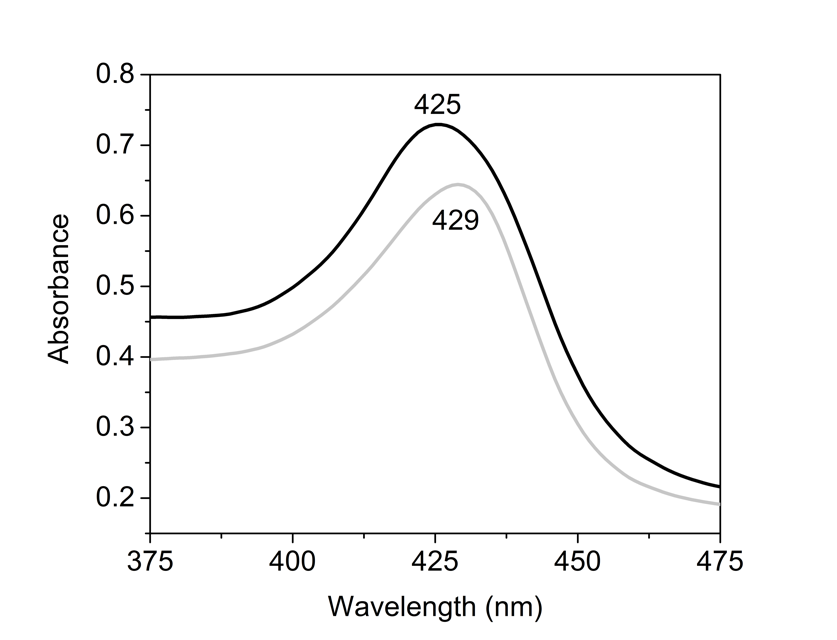

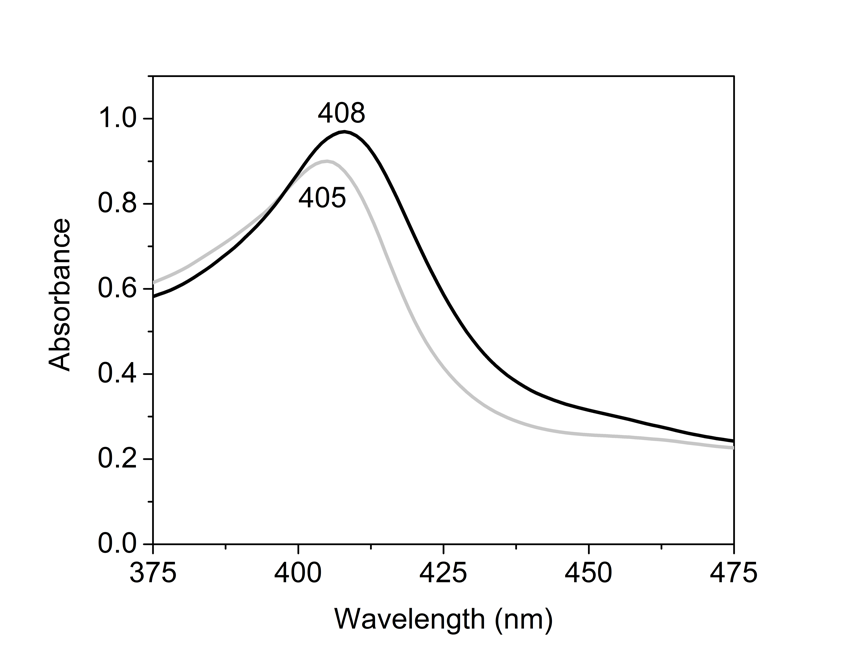


**Figure S9.** HmuW induced shift in Soret absorbance maxima. (A) Absorbance spectra of 10 μM Fe^2+^heme-HmuF (grey line) and with addition of 10 μM HmuW (black line). (B) Absorbance spectra of 10 μM Fe^3+^-heme-HmuF (grey line) and with the addition 10 μM HmuW (black line).

β3

⍺2

β2

⍺1

β1

fnu:FldH MKTLIIYSSET**GNT**KMVCEKAFEYINGEKVI-IPIKE-EDSINLDEFDNIVV**G**TWI**D**KAN 58

fnu:HmuF MKTLIVYSTIS**GNT**KSVCERIYGALNAEKEI-INVKD-IKNLQVNNYDNFII**G**FWC**D**KGT 58

fpd MKTLIVYSTIS**GNT**KAVCERIYNALNVEKEI-INVKD-SKNIKPSDYENIII**G**FWC**D**KGT 58

lhf MSSLVVFSTST**GNT**RKIADAIFSALKDTDKKIVDV-NEINTVNMNEFEKIII**G**GWI**D**KGE 59

lte MSSLVVFSTST**GNT**KKIADAIFSALKDTDKKIVDV-NEINKVNFNKFDKIII**G**GWI**D**KGE 59

lgo MSTLVVYSTLT**GNT**KKVAEAVFEAISGEKELKNVS-EVEKTENFEKFDKIIF**G**YWV**D**KGD 59

csph MRNLVIYASKT**GNT**KKVAMAIANSLGYELIS---V-DE--IENLNGVNNIIF**G**FYI**D**KGD 54

cure MKTLVLYHSLT**GNT**KKVATAIAKSKNADLKS---I-DE--EFDLDNYECVIF**G**FYV**D**RGF 54

ccv MKKIVIYASLS**GNT**KKIGEAIAQQIGCEAIS---Y-TDERAKDISGFDFVAI**G**FYI**D**KGG 56

cgra MKKIVLYTSQT**GNT**KKVGDAIAEQLGCKSLN---F-R-DFEGEVDDYDFIAL**G**FYV**D**KGE 55

csho MKKIVIYTSAT**GNT**EKVGLAIANELGCEAVK---F-SEDLHLDLDRYDFIAL**G**FYV**D**KGD 56

vat MGTIILYSSLT**GNT**KSVAEAMASVMPEGTPC-VPVK--EAPANLGDYDTVFL**G**FWV**D**RGT 57

fva MKTLVTYSTKT**GNT**KKVAESIAKAIKNSEI--MDISEVK----NLDYDLIII**G**TWI**D**KGT 54

tde MKILLTYSSKT**GNT**KAVAEAVLKTLPQGT-DFFAVSE---VKDVNNYDAVIV**G**FWI**D**KGL 56

⍺4

β5

⍺3

β4

fnu:FldH ANAEARKFIN-TLSNKKIFFIG**T**LAASLESEHAKKCFNNLTKLCSKKNN-FVDGVLTR**G**K 116

fnu:HmuF MDKDSIDFLK-ILNNKNIYFVG**T**LGADPGSGHWNDVFENAKKLCSENNN-FKDGLLIW**G**R 116

fpd MDKDSIDFLK-TLNNKNLYFLG**T**LGARPDSEHWNDVFENAKKLCSENNI-FKDGLLIW**G**R 116

lhf IDEKSKEFLT-NLKNKKLGLFV**T**MGGNPETDRAKNCFQEIKKSLEKNGNIVEKTFVCQ**G**A 118

lte IDEKAKEFLT-KLKNKKLGLFV**T**MGGNPETDRAKNCFQEIKKSLEKNGNIVEKIFVCQ**G**A 118

lgo ADERMKKFMA-KVKNKTVGAFG**T**LGAKPDSDHAKRCLEKVKTFLEENGNKVEREFICR**G**A 118

cspf MSDNAKRVAL-MIKNRKIGLFM**T**LGASANSDHAKECFSKAKDEFIKNGCEIGGEFFCQ**G**A 113

cure FVPKAENVAK-NIKNKTMGLFF**T**LGAEPDGDHAKDCEKKALDYFTKLGNNVKATFCCQ**G**A 113

ccv PEAHFKRYIKEHVKGKKTGLFI**T**LGADPAGEHGESMLKVGRELLNEGGNEILAEFICQ**G**A 116

cgra AEAKFTRFIR-KIHGKKLGVFM**T**LGAEPDGEHSRKCLDTFEEGLKANGNEIIREFACQ**G**A 114

csho AEPKFKRFLR-EIKGKKVGLFM**T**LGMDPEHEHAMNCLEKAKVVLREGENEILREFYCQ**G**A 115

vat ANKEAAKLIE-TLTNKHIVFFA**T**LGMYADSDHARESIEKASALLP-NKESLVDGFVCQ**G**K 115

fva ADAKALNFIK-TLANKNTAFFF**T**LGAYPDSKHALDCVENITKLFTDNENKVLGHFLCQ**G**A 113

tde PNEEALNFME-TIKNKKTGYFF**T**LGAYPDSPHAEDCHKSAKELLTKNGNEVLAGFGCQ**G**K 115

⍺8

⍺7

⍺6

⍺5

fnu:FldH VSKDLQEKFTK----FPLNII**H**KFVPNMKEIILE**A**DC**HP**NES**D**FLLIKGFIDKNFNY--- 169

fnu:HmuF ISQEMQDMMKN----FPASHP**H**AVTPERLARWEA**A**ST**HP**DEN**D**FKKAEEFFSNLLNN--- 169

fpd ISKEMMDVMKK----FPAGHP**H**AVTPERLARWEA**A**ST**HP**DEN**D**FKKAEEFFSNLLNK--- 169

lhf IDPNLINKFREMTKQG-IAGP**F**AVTPEREARWAE**A**AK**HP**DEK**D**IENAKRIFGGL------ 171

lte IDPNLINKFREMTKQG-IAGP**F**AATPEREARWAE**A**VK**HP**DEK**D**IENAKRIFGGL------ 171

lgo IDPKLLDKFRKMTAEG-MTGH**H**AATPEAEKRWAE**A**AK**HP**NEE**D**FENAKRAFEGF------ 171

cspf IDPELIAWMRA---NLG----**D**KITPQKEEGWKN**A**AS**HP**DKE**D**LKNAVLAFMDFN----- 161

cure IDPKVIEQMRQMAAKMGDKAV**H**QITPQREARWKR**A**AS**HP**DEN**D**LKNAVLAFESL------ 167

ccv IDPKVIEEMKEMAAKMGDKAI**H**LITPERQATWKQ**A**ST**HP**DEN**D**LKNARKAFEGIK----- 171

cgra IDPNLLETMRKMASSG--NSP**H**LITPERLARWAE**A**AK**HP**DEK**D**LADAKAAFAGIE----- 167

csho IDPKVIEQLRKMGEAAPNDPR**Y**AVTPEREARWAR**A**AT**HP**DAN**D**LENAKAVFKGI------ 169

vat IDPKVIEMMYKM---FPPGSA**H**GQSPERDALHKA**A**ET**HP**DEQ**D**FANAKEFAKSVLAKLQG 172

fva VDPKLIEMMKTK---LGPDHP**H**GPNPERIKRWAD**A**SL**HP**DET**D**LNNAYVYFKELVEKL-- 168

tde IDPALTEMFKS----LPKDHP**H**YMNEERRKRHEE**A**AK**HP**DKK**D**FENAKKAFENFGR---- 167

**Figure S10. Protein sequence alignment of HmuF homologs from different Gram-negative bacteria.** The alignment was performed by Clustal MUSCLE. The homologs were selected based on if the associated annotated flavodoxin gene was present in a heme uptake/utilization cluster, neighboring a gene encoding an anaerobilin synthase with >40 % sequence identity to HmuW. The gene locus tag for each flavodoxin is written in parenthesis. The KEGG organism codes are as follows: Fnu, *Fusobacterium nucleatum* subsp. *nucleatum*; fpd, *Fusobacterium pseudoperidonticum* (CTM68_10175); lfh, *Leptotrichia hofstadii* JCM16775_0692); lte, *Leptotrichia trevisanii* (JMUB4039_1844); lgo, *Leptotrichia buccalis* (JCM16774_1763); cspf, *Campylobacter sputorum,* (CSF_0146); cure, *Campylobacter* *ureolyticus* (CUREO_0227); ccv. *Campylobacter curvus* (CCV52592_0646), cgra; *Campylobacter* *gracillis*; (CGRAC_0444); csho, *Campylobacter showae* (CSHOW_1425)*;* vat, *Veillonella atypica* (B7L28_06770); fva, *Fusobacterium varium* (FV113G1_19080), tde, *Trepodema denticola* (TDE_1177)*.* The gene identifier is listed after the colon. The location of secondary structural elements in FldH are shown (orange boxes ⍺-helices and blue arrows, β-strands). The dark orange boxes denote the ⍺-helices found in the helical cap domain. Those residues aligned with H134 of FldH are shown in bold. Hydrophobic residues that made van der Waals contact with the heme are denoted with green circles and residues interacting with the FMN are identified with red circles. Invariant residues are blue (bold type).
